# Impact of left ventricular ejection fraction on the effect of renin-angiotensin system blockers after an episode of acute heart failure: From the KCHF Registry

**DOI:** 10.1371/journal.pone.0239100

**Published:** 2020-09-14

**Authors:** Yusuke Yoshikawa, Yodo Tamaki, Takeshi Morimoto, Hidenori Yaku, Erika Yamamoto, Yasutaka Inuzuka, Neiko Ozasa, Takeshi Kitai, Kazuya Nagao, Yukihito Sato, Hirokazu Kondo, Toshihiro Tamura, Yoshihisa Nakagawa, Koichiro Kuwahara, Takao Kato, Takeshi Kimura

**Affiliations:** 1 Department of Cardiovascular Medicine, Kyoto University Graduate School of Medicine, Kyoto, Japan; 2 Department of Cardiology, Tenri Hospital, Tenri, Japan; 3 Department of Clinical Epidemiology, Hyogo College of Medicine, Nishinomiya, Japan; 4 Department of Cardiology, Mitsubishi Kyoto Hospital, Kyoto, Japan; 5 Department of Cardiovascular Medicine, Shiga General Hospital, Moriyama, Japan; 6 Department of Cardiovascular Medicine, Kobe City Medical Center General Hospital, Kobe, Japan; 7 Division of Cardiology, Osaka Red Cross Hospital, Osaka, Japan; 8 Department of Cardiology, Hyogo Prefectural Amagasaki General Medical Center, Amagasaki, Japan; 9 Division of Cardiovascular Medicine, Shiga University of Medical Science, Otsu, Japan; 10 Department of Cardiovascular Medicine, Shinshu University, Matsumoto, Japan; Scuola Superiore Sant'Anna, ITALY

## Abstract

**Objective:**

This observational study aimed to examine the prognostic association of angiotensin-converting enzyme inhibitors (ACE-I)/angiotensin receptor blockers (ARB) in different left ventricular ejection fraction (LVEF) categories.

**Methods:**

In 3717 patients enrolled in the KCHF Registry, a multicentre registry including consecutive patients hospitalized for acute heart failure (HF), we assessed patient characteristics and association between ACE-I/ARB and clinical outcomes according to LVEF. In the three LVEF categories (reduced LVEF [HFrEF], mid-range LVEF [HFmrEF] and preserved LVEF [HFpEF]), we compared the patients with ACE-I/ARB as discharge medication and those without, and assessed their 1-year clinical outcomes. We defined the primary outcome measure as a composite of all-cause death and HF hospitalization.

**Results:**

The 1-year cumulative incidences of the primary outcome measure were 36.3% in HFrEF, 30.1% in HFmrEF and 33.8% in HFpEF (log-rank P = 0.07). The adjusted risks of the ACE-I/ARB group relative to the no ACE-I/ARB group for the primary outcome measure were significantly lower in HFrEF and HFmrEF (HR 0.66 [95%CI 0.54–0.79], P<0.001, and HR 0.61 [0.45–0.82], P = 0.001, respectively), but not in HFpEF (HR 0.95 [0.80–1.14], P = 0.61). There was a significant interaction between the LVEF category and the ACE-I/ARB use on the primary outcome measure (P_interaction_ = 0.01).

**Conclusions:**

ACE-I/ARB for patients who were hospitalized for acute HF was associated with significantly lower risk for a composite of all-cause death and HF hospitalization in HFrEF and HFmrEF, but not in HFpEF. ACE-I/ARB might be a potential treatment option in HFmrEF as in HFrEF.

## Introduction

Previous randomized clinical trials have shown the prognostic and symptomatic benefits of angiotensin-converting enzyme inhibitors (ACE-I) and angiotensin receptor blockers (ARB) in heart failure with reduced ejection fraction (HFrEF) [1–5]. On the other hand, a substantial proportion of the patients with heart failure (HF) have left ventricular ejection fraction (LVEF) higher than the reduced-LVEF range [6–8]. Studies focusing on renin-angiotensin system (RAS) antagonists failed to prove definitive effectiveness in improving prognosis and symptoms of these patients [9–11].

The ESC 2016 guideline recognizes the evidence gap between HFrEF and HF with preserved ejection fraction (HFpEF) [12], because the previous trials enrolling patients with LVEF higher than reduced-LVEF range defined various cutoff values (40%, 45%, 50%) [13]. For instance, I-PRESERVE [11] enrolled subjects with LVEF ≥45% and PEP-CHF [10] included elderly patients with left ventricular wall motion index of 1.4–1.6, corresponding to LVEF approximately ≥40%. In this context, it has defined a new category of HF with mid-range ejection fraction (HFmrEF, LVEF 40–49%) along with HFrEF (LVEF <40%) and HFpEF (LVEF ≥50%) [12]. In the current guidelines [12, 14], the treatment for HFmrEF is described in line with that for HFpEF because LVEF ≥40% has generally been categorized as preserved-LVEF. Thus, no formal treatment except diuretics to alleviate congestion is recommended for these categories at present, although recent reports have indicated the effectiveness of RAS inhibitors in HFmrEF as well as HFrEF [15–17].

It would be intriguing to examine the treatment effect of RAS inhibitors in a large observational registry including all spectrum of LVEF, although large randomized controlled trials dedicated to HFmrEF are desirable [18, 19]. This study aimed to examine the association of ACE-I/ARB with clinical outcomes in different LVEF categories and their interactions in the multicenter observational cohort study in Japan.

## Methods

### Study population and data collection

The Kyoto Congestive Heart Failure registry is a physician-initiated, prospective, observational, multicenter cohort study enrolling the consecutive patients who were hospitalized for acute HF between October 2014 and March 2016 at 19 secondary and tertiary hospitals in Japan.

The study was approved by the institutional review boards of Kyoto University Graduate School of Medicine (approval number: E2311); Shiga General Hospital (approval number: 20141120–01); Tenri Hospital (approval number: 640); Kobe City Medical Center General Hospital (approval number: 14094); Hyogo Prefectural Amagasaki General Medical Center (approval number: Rinri 26–32); National Hospital Organization Kyoto Medical Center (approval number: 14–080); Mitsubishi Kyoto Hospital (approved 11/12/2014); Okamoto Memorial Hospital (approval number: 201503); Japanese Red Cross Otsu Hospital (approval number: 318); Hikone Municipal Hospital (approval number: 26–17); Japanese Red Cross Osaka Hospital (approval number: 392); Shimabara Hospital (approval number: E2311); Kishiwada City Hospital (approval number: 12); Kansai Electric Power Hospital (approval number: 26–59); Shizuoka General Hospital (approval number: Rin14-11-47); Kurashiki Central Hospital (approval number: 1719); Kokura Memorial Hospital (approval number: 14111202); Kitano Hospital (approval number: P14-11-012); and Japanese Red Cross Wakayama Medical Center (approval number: 328). A waiver of written informed consent from each patient was granted by the institutional review boards of Kyoto University and each participating centre based on the Japanese guidelines for epidemiological study [20]. All the data were fully anonymized before we accessed them. The investigation conforms with the principles outlined in the Declaration of Helsinki.

The details of the design and patient enrollment have been described previously [8, 21, 22]. In brief, we consecutively enrolled the patients who had the acute HF symptoms defined by the modified Framingham criteria and who received treatment for heart failure involving intravenous drugs (e.g. diuretics, vasodilators and inotropes) within 24 hours after hospital presentation. We anonymized the patient data before analysis. When a value in the data was out of the expected range, the attending investigators rechecked the hospital charts for the value. The patients in this study were censored in October 2017 and we performed time-to-event analyses. The data were collected from the hospital charts by attending physicians and/or clinical research assistants at each participating hospital. When patients were lost to follow-up, the attending physicians obtained the additional information by phone and/or mail to patients, relatives, and/or referring physicians. The duration of follow-up of the patients who survived for at least 1 year was 522 days [396–682] (median [interquartile ranges]).

### Definitions

The detailed definitions of baseline patient characteristics were described previously [8] and in the S1 File. We defined the use of ACE-I/ARB as any prescription of ACE-I and/or ARB at discharge of the index hospitalization.

The primary outcome measure in the current analysis was a composite of all-cause death and HF hospitalization during follow-up [10, 11, 22]. The secondary outcome measures were individual components of the primary outcome measure and cardiovascular death during the follow-up. Death was regarded as cardiovascular in origin unless obvious non-cardiovascular causes could be identified. Cardiovascular death included HF-related death, sudden death, stroke-related death and any other cardiovascular death. Sudden death was defined as unexplained death in patients who were stable until death. Stroke was defined as ischaemic or hemorrhagic stroke requiring hospitalization with symptoms lasting >24 hours. HF hospitalization was defined as hospitalization due to worsening HF requiring intravenous drug therapy. Clinical outcomes were assessed by multiple investigators; when there was disagreement, the attending physicians rechecked hospital charts for the event. LVEF was classified according to the baseline data; HFrEF was defined as LVEF <40%, HFmrEF as LVEF 40–49%, HFpEF as LVEF ≥50% [12]. Laboratory data were obtained at the time of admission.

### Statistical analysis

We summarized the baseline patient characteristics across the three LVEF categories and compared the ACE-I/ARB group to the no ACE-I/ARB group in each LVEF stratum. Categorical variables were presented as numbers and percentages, and continuous variables were presented as mean and standard deviation (SD) or median and 25th to 75th percentiles according to their distributions. Categorical variables were compared by the chi-squared test when appropriate; otherwise, by the Fisher’s exact test. Continuous variables were compared by Student's t test (or the one-way analysis of variance) or Wilcoxon rank sum test (or Kruskal-Wallis test) based on their distributions.

In each LVEF stratum, we compared the patients with ACE-I/ARB at discharge to those without ACE-I/ARB for the clinical outcomes during the follow-up. Cumulative incidences were estimated by the Kaplan-Meier method and the differences were assessed by the log-rank test. We constructed Cox proportional hazard models to estimate hazard ratios (HR) and their 95% confidence intervals (CI) of the ACE-I/ARB group relative to the no ACE-I/ARB group for each clinical outcome measure. To adjust for confounders, we incorporated the 24 clinically relevant factors listed in Table 1 along with use of ACE-I/ARB into multivariable Cox proportional hazard models in line with our previous reports [22, 23]. For multivariable analysis, missing data of binary variables were considered as negative values. The interactions between the LVEF category and the use of ACE-I/ARB were also examined with the product terms in the Cox models. Additionally, we conducted a sensitivity analysis using propensity-score (PS) matching analysis in each LVEF stratum.

**Table 1:** Baseline patient characteristics stratified by the LVEF category.

	HFrEF		HFmrEF		HFpEF		
	N = 1383		N = 703		N = 1631		P value
Age [years]	77	[66–84]	80	[72–86]	82	[76–88]	<0.001
Age ≥80*	549	(40%)	366	(52%)	1013	(62%)	<0.001
Women*	458	(33%)	283	(40%)	927	(57%)	<0.001
BMI [kg/m2]	22.9	±4.6	22.7	±4.2	23.0	±4.4	0.43
BMI <22*	628	(47%)	310	(47%)	700	(46%)	0.73
Etiology							
Chronic CAD	620	(45%)	289	(41%)	296	(18%)	<0.001
Acute coronary syndrome*	86	(6.2%)	55	(7.8%)	64	(3.9%)	<0.001
Hypertensive heart disease	184	(13%)	179	(26%)	564	(35%)	<0.001
Cardiomyopathy	397	(29%)	71	(10%)	88	(5.4%)	<0.001
Valvular heart disease	134	(9.7%)	133	(19%)	468	(29%)	<0.001
Medical history							
HF hospitalization*	531	(39%)	237	(34%)	549	(34%)	0.008
AF/AFL*	438	(32%)	292	(42%)	820	(50%)	<0.001
Hypertension*	911	(66%)	536	(76%)	1243	(76%)	<0.001
Diabetes mellitus*	567	(41%)	286	(41%)	539	(33%)	<0.001
Dyslipidemia	582	(42%)	293	(42%)	577	(35%)	<0.001
Prior myocardial infarction*	442	(32%)	216	(31%)	178	(11%)	<0.001
Prior stroke*	190	(14%)	116	(17%)	284	(17%)	0.02
Prior PCI/CABG	434	(31%)	221	(31%)	298	(18%)	<0.001
Current smoking*	221	(16%)	101	(15%)	130	(8.1%)	<0.001
VT/VF	115	(8.3%)	18	(2.6%)	21	(1.3%)	<0.001
CRT	57	(4.1%)	9	(1.3%)	5	(0.3%)	<0.001
Lung disease*	170	(12%)	75	(11%)	243	(15%)	0.01
Cancer	180	(13%)	104	(15%)	251	(15%)	0.17
Dementia	205	(15%)	119	(17%)	331	(20%)	<0.001
Social backgrounds							
On job	271	(20%)	91	(13%)	132	(8.1%)	<0.001
Living alone	308	(22%)	150	(21%)	336	(21%)	0.54
Activities of daily living							
Ambulatory*	1144	(84%)	570	(82%)	1227	(76%)	<0.001
Wheelchair	176	(13%)	110	(16%)	324	(20%)	
Bedridden	48	(3.5%)	17	(2.4%)	64	(4.0%)	
Vital signs at presentation							
Systolic BP [mmHg]	142.9	±33.1	153.2	±35.7	150.8	±35.7	<0.001
Systolic BP <90*	41	(3.0%)	12	(1.7%)	42	(2.6%)	0.23
Diastolic BP [mmHg]	88.3	±23.6	87.5	±23.9	81.3	±23.6	<0.001
Heart rate [/min]	101	±25.3	99.3	±28.3	90.2	±28.3	<0.001
Heart rate <60	34	(2.5%)	37	(5.3%)	179	(11%)	<0.001
NYHA class III or IV*	1213	(88%)	606	(87%)	1403	(86%)	0.27
LVEF [%]	29.1	±7.1	44.3	±2.9	61.9	±7.5	NA
Laboratory tests at admission							
BNP [pg/ml]	950	[580–1634]	783	[451–1281]	491	[281–877]	<0.001
NT-proBNP [pg/ml]	7078	[3218–14576]	6355	[2820–18328]	4716	[2214–9612]	<0.001
Blood urea nitrogen [mg/dl]	23.6	[17.8–34.5]	24.7	[17.8–34.0]	23.8	[17.4–34.0]	0.76
Creatinine [mg/dl]	1.12	[0.86–1.62]	1.10	[0.82–1.73]	1.07	[0.80–1.53]	0.005
Creatinine ≥2	221	(16%)	138	(20%)	239	(15%)	0.01
eGFR [ml/min/1.73m^2^]	45.7	[30.4–61.5]	44.7	[26.6–62.2]	43.3	[29.0–59.2]	0.052
eGFR <30*	337	(25%)	204	(29%)	437	(27%)	0.07
Albumin [g/dl]	3.53	±0.47	3.46	±0.50	3.46	±0.49	<0.001
Albumin <3*	149	(11%)	101	(15%)	230	(15%)	0.01
Sodium [mEq/l]	139.0	±4.2	139.2	±4.3	139.3	±4.1	0.15
Sodium <135*	166	(12%)	74	(11%)	193	(12%)	0.59
Potassium [mEq/l]	4.2	±0.7	4.2	±0.6	4.2	±0.7	0.65
Potassium ≥5.0*	170	(12%)	77	(11%)	185	(11%)	0.59
Haemoglobin [g/dl]	12.3	±2.4	11.5	±2.3	11.0	±2.2	<0.001
Anemia*	766	(56%)	471	(67%)	1220	(75%)	<0.001
ACE-I/ARB at admission*	616	(45%)	307	(44%)	775	(48%)	0.13
Medications at discharge							
ACE-I/ARB	892	(65%)	400	(57%)	846	(52%)	<0.001
Both ACE-I and ARB	7	(0.5%)	1	(0.1%)	9	(0.6%)	0.41
ACE-I	481	(35%)	158	(23%)	270	(17%)	<0.001
ARB	418	(30%)	243	(35%)	585	(36%)	0.004
MRA*	722	(52%)	310	(44%)	646	(40%)	<0.001
β-blockers*	1080	(78%)	504	(72%)	885	(54%)	<0.001
Loop diuretics*	1146	(83%)	560	(80%)	1309	(80%)	0.11
Thiazide	63	(4.6%)	36	(5.1%)	119	(7.3%)	0.004
Tolvaptan	169	(12%)	56	(8.0%)	165	(10%)	0.009
Digoxin	88	(6.4%)	30	(4.3%)	93	(5.7%)	0.15
Warfarin	329	(24%)	155	(22%)	440	(27%)	0.02
DOAC	227	(16%)	153	(22%)	380	(23%)	<0.001

*Risk-adjusting variables for the multivariable Cox regression model.

ACE-I, angiotensin-converting-enzyme inhibitors; AF, atrial fibrillation; AFL, atrial flutter; ARB, angiotensin receptor blockers; BMI, body mass index; BNP, brain natriuretic peptide; BP, blood pressure; BUN, blood urea nitrogen; CABG, coronary artery bypass grafting; CAD, coronary artery disease; DOAC, direct oral anticoagulants; eGFR, estimated glomerular filtration rate; HF, heart failure; HFmrEF, heart failure with mid-range ejection fraction; HFpEF, heart failure with preserved ejection fraction; HFrEF, heart failure with reduced ejection fraction; LVEF, left ventricular ejection fraction; MRA, mineralocorticoid receptor antagonists; NT-proBNP, N-terminal pro-B-type natriuretic peptide; NYHA, New York Heart Association; PCI, percutaneous coronary intervention; VF, ventricular fibrillation; VT, ventricular tachycardia.

A physician (Y.Y.) performed the statistical analysis with R version 3.6.3 (R Foundation for Statistical Computing, Vienna, Austria). The reported P values were two-sided, and P values <0.05 were considered statistically significant.

## Results

### Patient characteristics

Among 4056 patients involved in the KCHF registry, 3785 patients survived to discharge during the index hospitalization. In the present study, we sought to compare the clinical outcomes between the patients who received ACE-I/ARB at discharge and those who did not. To stratify them by the LVEF category, we excluded 11 patients whose baseline LVEF data at the index hospitalization was missing. After exclusion of 57 patients without any follow-up data after discharge, the current study population consisted of 3717 patients hospitalized for acute HF and known LVEF who were discharged alive from the index hospitalization.

**Fig 1:**
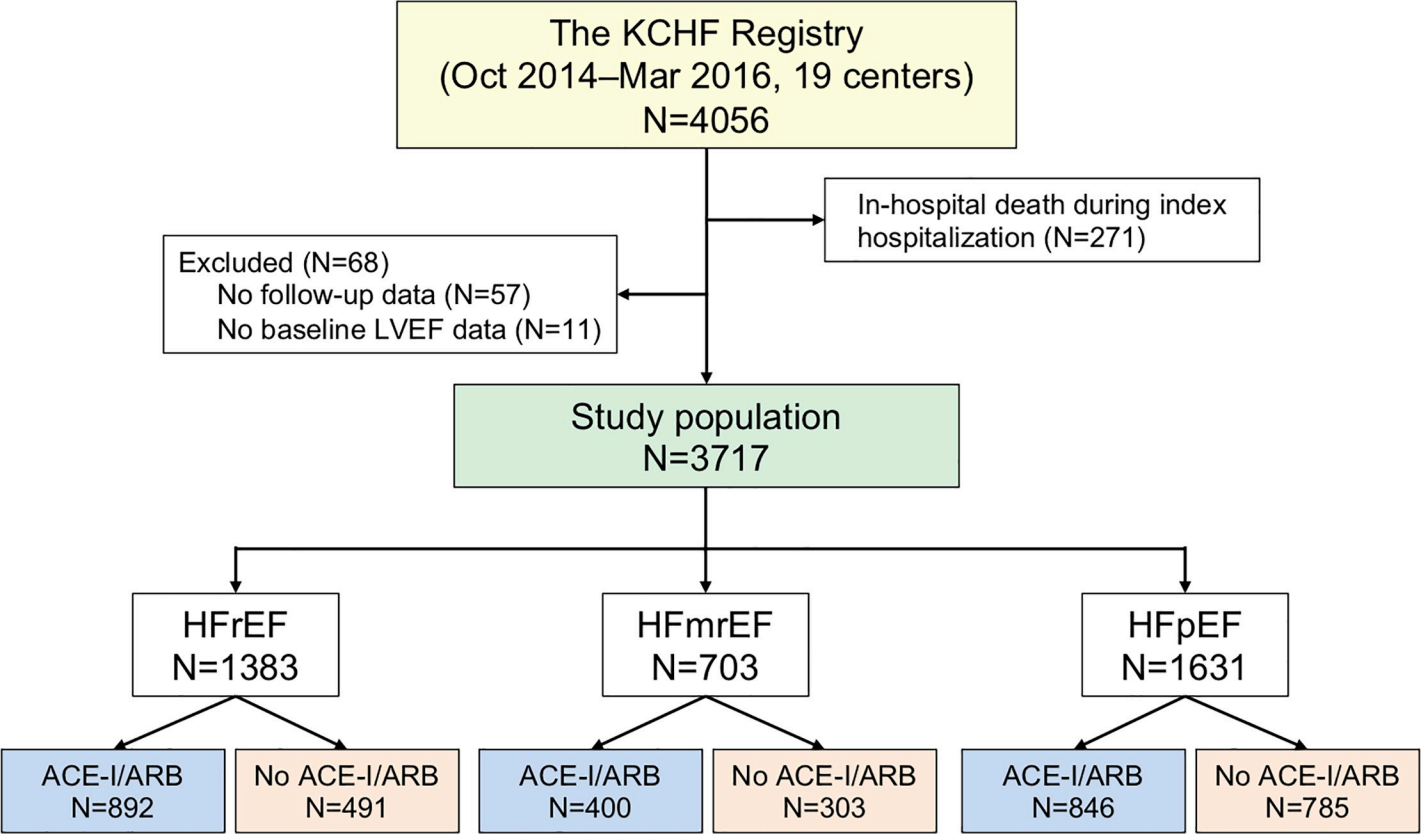
Study flowchart. A total of 3717 patients were included in this study from the KCHF Registry. ACE-I, angiotensin-converting enzyme inhibitors; ARB, angiotensin receptor blockers; HFmrEF, heart failure with mid-range ejection fraction; HFpEF, heart failure with preserved ejection fraction; HFrEF, heart failure with reduced ejection fraction; LVEF, left ventricular ejection fraction.

Among the entire population of 3717 patients, there were 1383 patients (37%) with HFrEF, 703 (19%) with HFmrEF and 1631 (44%) with HFpEF (Fig 1). The patient characteristics stratified by the LVEF category and missing values were summarized (Table 1, S1 Table). Compared with the HFrEF group, the patients in the HFpEF group were older, included higher proportions of women, had lower activity of daily living and a higher prevalence of valvular etiology, hypertension, atrial arrythmia, and dementia than those in the HFrEF group, while the patients in the HFrEF group were more likely to have coronary artery disease and worse renal function and were more often prescribed ACE-I/ARB, mineralocorticoid receptor antagonists and β-blockers than those in the HFpEF group. The patients in the HFmrEF group in general had intermediate patient characteristics between the HFrEF and HFpEF groups. However, the patients in both the HFmrEF and HFrEF groups had a much higher prevalence of coronary artery disease than those in the HFpEF group.

ACE-I/ARB was prescribed for 65% of the patients in the HFrEF group, 57% of those in the HFmrEF group and 52% of those in the HFpEF group (Fig 1). The types and doses of ACE-I/ARB were summarized in S2 Table. Across the three LVEF groups, those with ACE-I/ARB at discharge compared to those without ACE-I/ARB were younger and more likely to have hypertension, higher blood pressure at presentation and better activity of daily living. Conversely, those without ACE-I/ARB tended to have higher prevalence of malignancy and dementia, and hyperkalemia at presentation (Table 2). Among the patients prescribed ACE-I/ARB, uptitration of ACE-I/ARB was accomplished in 20% of the HFrEF group, 25% of the HFmrEF group and 32% of the HFpEF group, respectively (S1 Fig). Patients who were younger, had better renal function and higher blood pressure were more likely to be prescribed ACE-I/ARB (S2 Fig).

**Table 2:** Baseline characteristics stratified by the LVEF categories: ACE-I/ARB versus no ACE-I/ARB.

	HFrEF					HFmrEF					HFpEF				
	ACE-I/ARB		No ACE-I/ARB			ACE-I/ARB		No ACE-I/ARB			ACE-I/ARB		No ACE-I/ARB		
	N = 892		N = 491		P value	N = 400		N = 303		P value	N = 846		N = 785		P value
Age [years]	74	[63–82]	80	[72–86]	<0.001	79	[72–85]	82	[72–87]	0.03	82	[75–87]	83	[76–88]	0.002
Age ≥80*	302	(34%)	247	(50%)	<0.001	195	(49%)	171	(56%)	0.043	502	(59%)	511	(65%)	0.02
Women*	260	(29%)	198	(40%)	<0.001	153	(38%)	130	(43%)	0.21	479	(57%)	448	(57%)	0.85
BMI [kg/m2]	23.4	±4.7	21.9	±4.3	<0.001	23.1	±4.5	22.1	±3.9	0.004	23.4	±4.7	22.5	±4.1	<0.001
BMI <22	381	(44%)	247	(52%)	0.003	165	(43%)	145	(51%)	0.043	346	(43%)	354	(49%)	0.02
Etiology															
Chronic CAD	384	(43%)	236	(48%)	0.07	166	(42%)	123	(41%)	0.81	170	(20%)	126	(16%)	0.03
Acute coronary syndrome*	56	(6.3%)	30	(6.1%)	0.90	36	(9.0%)	19	(6.3%)	0.18	39	(4.6%)	25	(3.2%)	0.14
Hypertensive heart disease	131	(15%)	53	(11%)	0.041	115	(29%)	64	(21%)	0.02	343	(41%)	221	(28%)	<0.001
Cardiomyopathy	283	(32%)	114	(23%)	0.001	36	(9.0%)	35	(12%)	0.27	41	(4.8%)	47	(6.0%)	0.31
Valvular heart disease	70	(7.8%)	64	(13%)	0.002	70	(18%)	63	(21%)	0.27	214	(25%)	254	(32%)	0.002
Medical history															
HF hospitalization*	319	(37%)	212	(44%)	0.007	127	(32%)	110	(37%)	0.19	277	(33%)	272	(36%)	0.29
AF/AFL*	269	(30%)	169	(34%)	0.10	155	(39%)	137	(45%)	0.09	403	(48%)	417	(53%)	0.03
Hypertension*	605	(68%)	306	(62%)	0.04	324	(81%)	212	(70%)	0.001	730	(86%)	513	(65%)	<0.001
Diabetes mellitus*	370	(42%)	197	(40%)	0.62	156	(39%)	130	(43%)	0.30	321	(38%)	218	(28%)	<0.001
Dyslipidemia	380	(43%)	202	(41%)	0.60	173	(43%)	120	(40%)	0.33	333	(39%)	244	(31%)	<0.001
Prior myocardial infarction*	270	(30%)	172	(35%)	0.07	128	(32%)	88	(29%)	0.40	105	(12%)	73	(9.3%)	0.044
Prior stroke*	106	(12%)	84	(17%)	0.007	70	(18%)	46	(15%)	0.41	145	(17%)	139	(18%)	0.76
Prior PCI/CABG	268	(30%)	166	(34%)	0.15	129	(32%)	92	(30%)	0.59	181	(21%)	117	(15%)	0.001
Current smoking*	171	(19%)	50	(10%)	<0.001	66	(17%)	35	(12%)	0.08	79	(9.5%)	51	(6.6%)	0.03
VT/VF	74	(8.3%)	41	(8.4%)	0.97	10	(2.5%)	8	(2.6%)	0.91	8	(0.9%)	13	(1.7%)	0.20
CRT	42	(4.7%)	15	(3.1%)	0.14	5	(1.2%)	4	(1.3%)	0.94	3	(0.4%)	2	(0.3%)	0.72
Lung disease*	105	(12%)	65	(13%)	0.43	41	(10%)	34	(11%)	0.68	130	(15%)	113	(14%)	0.58
Cancer	99	(11%)	81	(17%)	0.004	52	(13%)	52	(17%)	0.12	144	(17%)	107	(14%)	0.058
Dementia	97	(11%)	108	(22%)	<0.001	60	(15%)	59	(20%)	0.12	164	(19%)	167	(21%)	0.34
Social backgrounds															
On job	216	(24%)	55	(11%)	<0.001	61	(15%)	30	(9.9%)	0.036	77	(9.1%)	55	(7.0%)	0.12
Living alone	215	(24%)	93	(19%)	0.03	86	(22%)	64	(21%)	0.90	188	(22%)	148	(19%)	0.09
Activities of daily living															
Ambulatory*	772	(87%)	372	(77%)	<0.001	341	(86%)	229	(76%)	0.002	664	(79%)	563	(73%)	0.001
Wheelchair	83	(9.4%)	93	(19%)		51	(13%)	59	(20%)		156	(19%)	168	(22%)	
Bedridden	29	(3.3%)	19	(3.9%)		5	(1.3%)	12	(4.0%)		20	(2.4%)	44	(5.7%)	
Vital signs at presentation															
Systolic BP [mmHg]	146.8	±33.4	135.9	±31.6	<0.001	158.5	±36.3	146.3	±33.7	<0.001	158.5	±34.6	142.5	±34.9	<0.001
Systolic BP <90*	19	(2.1%)	22	(4.5%)	0.01	6	(1.5%)	6	(2.0%)	0.63	7	(0.8%)	35	(4.5%)	<0.001
Diastolic BP [mmHg]	91.1	±23.9	83.4	±22.3	<0.001	90.4	±24.6	83.6	±22.4	<0.001	83.8	±24.1	78.7	±22.9	<0.001
Heart rate [/min]	102.1	±24.9	98.9	±26.0	0.02	99.1	±28.4	99.6	±28.2	0.82	89.4	±28.3	91.1	±28.2	0.22
Heart rate <60	15	(1.7%)	19	(3.9%)	0.01	23	(5.8%)	14	(4.7%)	0.52	92	(11%)	87	(11%)	0.86
NYHA class III or IV*	778	(87%)	435	(90%)	0.21	351	(88%)	255	(84%)	0.15	734	(87%)	669	(86%)	0.48
LVEF [%]	29.2	±7.0	28.8	±7.2	0.31	44.3	±2.9	44.2	±2.9	0.70	62.3	±7.7	61.5	±7.2	0.056
Laboratory tests at admission															
BNP [pg/ml]	935	[581–1582]	1035	[571–1711]	0.18	746	[431–1264]	867	[460–1294]	0.21	467	[282–819]	525	[281–916]	0.10
NT-proBNP [pg/ml]	5401	[2737–12449]	8162	[5294–19533]	0.002	6281	[2682–17556]	6494	[3382–20340]	0.66	4650	[2095–8878]	4865	[2475–10664]	0.69
BUN [mg/dl]	22	[17.0–30.5]	27.8	[19.1–43.7]	<0.001	22.1	[17.0–30.6]	27	[19.0–39.5]	<0.001	22.1	[17.0–31.5]	25.0	[18.0–36.8]	<0.001
Creatinine [mg/dl]	1.06	[0.83–1.48]	1.27	[0.94–1.92]	<0.001	1.05	[0.80–1.50]	1.25	[0.85–2.05]	<0.001	1.02	[0.79–1.43]	1.13	[0.82–1.68]	0.001
Creatinine ≥2	105	(12%)	116	(24%)	<0.001	58	(15%)	80	(26%)	<0.001	98	(12%)	141	(18%)	<0.001
eGFR [ml/min/1.73m2]	49.8	[34.7–64.5]	38.3	[23.9–54.5]	<0.001	48.4	[33.2–63.4]	37.5	[22.3–59.5]	<0.001	45.6	[31.4–60.1]	41.2	[26.8–58.8]	<0.001
eGFR <30*	161	(18%)	176	(36%)	<0.001	86	(22%)	118	(39%)	<0.001	187	(22%)	250	(32%)	<0.001
Albumin [g/dl]	3.6	±0.5	3.5	±0.5	<0.001	3.5	±0.5	3.4	±0.5	<0.001	3.5	±0.5	3.4	±0.5	0.001
Albumin <3*	85	(9.7%)	64	(14%)	0.03	44	(11%)	57	(19%)	0.003	108	(13%)	122	(16%)	0.11
Sodium [mEq/l]	139.3	±3.9	138.4	±4.6	<0.001	139.4	±4.4	138.9	±4.1	0.11	139.6	±4.1	139.0	±4.2	0.005
Sodium <135	86	(9.7%)	80	(16%)	<0.001	38	(9.5%)	36	(12%)	0.31	87	(10%)	106	(14%)	0.046
Potassium [mEq/l]	4.15	±0.62	4.33	±0.76	<0.001	4.15	±0.61	4.25	±0.68	0.037	4.15	±0.64	4.24	±0.71	0.008
Potassium ≥5.0*	77	(8.7%)	93	(19%)	<0.001	38	(9.5%)	39	(13%)	0.16	82	(9.7%)	103	(13%)	0.03
Haemoglobin [g/dl]	12.6	±2.3	11.6	±2.4	<0.001	11.9	±2.3	11.0	±2.2	<0.001	11.1	±2.2	10.8	±2.1	0.03
Anemia*	442	(50%)	324	(66%)	<0.001	241	(60%)	230	(76%)	<0.001	622	(74%)	598	(76%)	0.23
ACE-I/ARB at admission*	492	(55%)	124	(25%)	<0.001	242	(61%)	65	(22%)	<0.001	584	(69%)	191	(24%)	<0.001
Medications at discharge															
ACE-I	481	(54%)	0	(0.0%)	NA	158	(40%)	0	(0.0%)	NA	270	(32%)	0	(0.0%)	NA
ARB	418	(47%)	0	(0.0%)	NA	243	(61%)	0	(0.0%)	NA	585	(69%)	0	(0.0%)	NA
Both ACE-I and ARB	7	(0.8%)	0	(0.0%)	NA	1	(0.3%)	0	(0.0%)	NA	9	(1.1%)	0	(0.0%)	NA
MRA*	517	(58%)	205	(42%)	<0.001	187	(47%)	123	(41%)	0.10	347	(41%)	299	(38%)	0.23
β-blockers*	755	(85%)	325	(66%)	<0.001	310	(78%)	194	(64%)	<0.001	496	(59%)	389	(50%)	<0.001
Loop diuretics*	746	(84%)	400	(82%)	0.31	320	(80%)	240	(79%)	0.80	691	(82%)	618	(79%)	0.13
Thiazide	32	(3.6%)	31	(6.3%)	0.02	19	(4.8%)	17	(5.6%)	0.61	62	(7.3%)	57	(7.3%)	0.96
Tolvaptan	100	(11%)	69	(14%)	0.12	31	(7.8%)	25	(8.3%)	0.81	64	(7.6%)	101	(13%)	<0.001
Digoxin	55	(6.2%)	33	(6.7%)	0.69	14	(3.5%)	16	(5.3%)	0.25	45	(5.3%)	48	(6.1%)	0.49
Warfarin	210	(24%)	119	(24%)	0.77	69	(17%)	86	(28%)	<0.001	187	(22%)	253	(32%)	<0.001
DOAC	153	(17%)	74	(15%)	0.32	93	(23%)	60	(20%)	0.27	217	(26%)	163	(21%)	0.02

*Risk-adjusting variables for the multivariable Cox regression model.

ACE-I, angiotensin-converting-enzyme inhibitors; AF, atrial fibrillation; AFL, atrial flutter; ARB, angiotensin receptor blockers; BMI, body mass index; BNP, brain natriuretic peptide; BP, blood pressure; BUN, blood urea nitrogen; CABG, coronary artery bypass grafting; CAD, coronary artery disease; DOAC, direct oral anticoagulants; eGFR, estimated glomerular filtration rate; HF, heart failure; HFmrEF, heart failure with mid-range ejection fraction; HFpEF, heart failure with preserved ejection fraction; HFrEF, heart failure with reduced ejection fraction; LVEF, left ventricular ejection fraction; MRA, mineralocorticoid receptor antagonists; NT-proBNP, N-terminal pro-B-type natriuretic peptide; NYHA, New York Heart Association; PCI, percutaneous coronary intervention; VF, ventricular fibrillation; VT, ventricular tachycardia.

### Clinical outcomes in the long-term follow-up in the three LVEF categories

**Fig 2:**
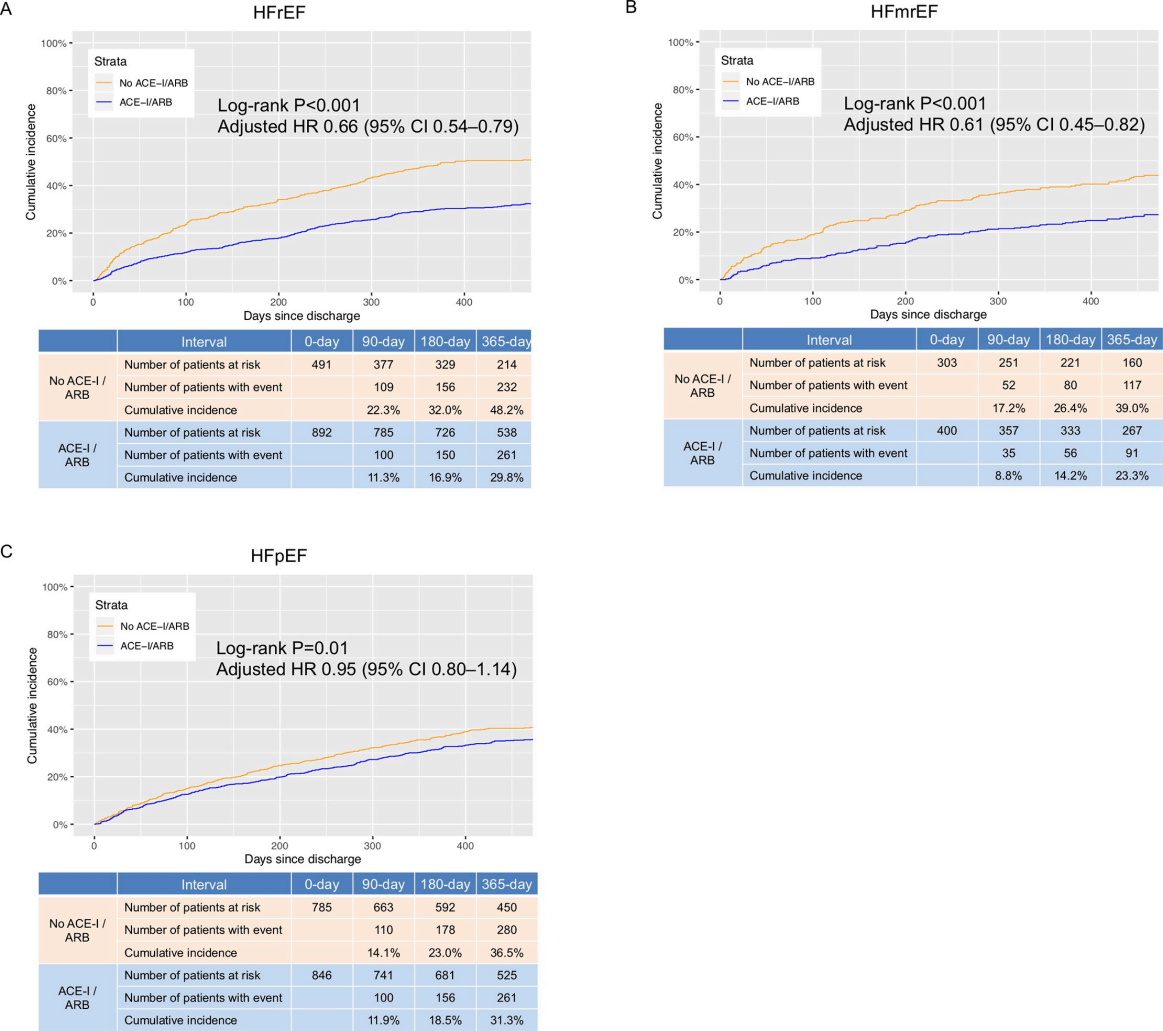
The Kaplan-Meier curves for the primary endpoint stratified by the LVEF category; ACE-I/ARB group versus no ACE-I/ARB group. The Kaplan-Meier curves represent the cumulative incidences of the primary outcome measure (a composite of all-cause death and HF hospitalization). (A) HFrEF, (B) HFmrEF, (C) HFpEF. LVEF, left ventricular ejection fraction; ACE-I, angiotensin-converting enzyme inhibitors; ARB, angiotensin receptor blockers; CI, confidence interval; HFmrEF, heart failure with mid-range ejection fraction; HFpEF, heart failure with preserved ejection fraction; HFrEF, heart failure with reduced ejection fraction; HR, hazard ratio.

We summarized cumulative incidences and adjusted HRs for long-term clinical outcomes stratified by the LVEF category (Table 3). The cumulative 1-year incidences of the primary outcome measure in the HFrEF, HFmrEF and HFpEF groups were 36.3%, 30.1% and 33.8%, respectively (log-rank P = 0.07, S3 Fig). In the three LVEF categories, the cumulative 1-year incidences of the primary outcome were lower in the ACE-I/ARB groups than in the no ACE-I/ARB groups (HFrEF, 29.8% versus 48.2%, log-rank P<0.001; HFmrEF, 23.3% versus 39.0%, log-rank P<0.001; 31.3% versus 36.5%, log-rank P = 0.01) (Fig 2). After adjusting for the confounders, the lower risks of the ACE-I/ARB groups relative to the no ACE-I/ARB groups for the primary outcome measure were significant in the HFrEF and HFmrEF categories (HR 0.66 [95%CI: 0.54–0.79], P<0.001, and HR 0.61 [95%CI: 0.45–0.82], P = 0.001, respectively), but not in the HFpEF category (HR 0.95 [95%CI: 0.80–1.14], P = 0.61) (Table 3). There was a significant interaction between the LVEF category, and the effect of ACE-I/ARB use at discharge on the primary outcome measure (P_interaction_ = 0.01). For each component of the primary outcome, the lower adjusted risks of the ACE-I/ARB groups relative to the no ACE-I/ARB groups were also significant for the individual components of the primary outcome measure in the HFrEF and HFmrEF groups (Table 3). There were trends toward interactions between the LVEF category, and the effect of ACE-I/ARB use at discharge on the components of the primary outcome measure (all cause death: P_interaction_ = 0.10, and HF hospitalization: P_interaction_ = 0.07), although those were not statistically significant.

**Table 3:** Effects of the use of ACE-I/ARB on clinical outcomes according to the LVEF category.

	ACE-I/ARB	No ACE-I/ARB	Crude HR (95% CI)	Adjusted HR (95% CI)	P value	P_interaction_
Primary outcome measure (a composite of all-cause death and HF hospitalization)						(LVEF*ACE-I/ARB)
HFrEF	261 (29.8%)	232 (48.2%)	0.53 (0.45–0.62)	0.66 (0.54–0.79)	<0.001	0.01
HFmrEF	91 (23.3%)	117 (39.0%)	0.54 (0.42–0.69)	0.61 (0.45–0.82)	0.001	
HFpEF	261 (31.3%)	280 (36.5%)	0.82 (0.71–0.96)	0.95 (0.80–1.14)	0.61	
All-cause death						
HFrEF	105 (12.1%)	130 (27.3%)	0.44 (0.35–0.56)	0.62 (0.48–0.81)	<0.001	0.10
HFmrEF	41 (10.5%)	76 (25.5%)	0.41 (0.30–0.57)	0.52 (0.35–0.77)	0.001	
HFpEF	114 (13.7%)	158 (20.7%)	0.64 (0.53–0.79)	0.73 (0.58–0.93)	0.01	
HF hospitalization						
HFrEF	200 (23.6%)	149 (33.8%)	0.63 (0.51–0.76)	0.73 (0.57–0.92)	0.009	0.07
HFmrEF	58 (15.4%)	67 (23.9%)	0.64 (0.46–0.88)	0.59 (0.40–0.87)	0.007	
HFpEF	187 (23.3%)	168 (23.9%)	0.98 (0.81–1.19)	1.14 (0.90–1.44)	0.28	
Cardiovascular death + HF hospitalization						
HFrEF	234 (27.1%)	192 (41.5%)	0.56 (0.47–0.67)	0.69 (0.56–0.85)	<0.001	0.07
HFmrEF	78 (20.4%)	90 (31.0%)	0.60 (0.46–0.79)	0.62 (0.44–0.88)	0.007	
HFpEF	217 (26.6%)	219 (29.6%)	0.86 (0.73–1.03)	0.99 (0.81–1.21)	0.92	
Cardiovascular death						
HFrEF	68 (8.0%)	87 (19.1%)	0.42 (0.32–0.56)	0.61 (0.44–0.84)	0.002	0.69
HFmrEF	27 (7.2%)	43 (15.2%)	0.45 (0.30–0.69)	0.52 (0.31–0.86)	0.01	
HFpEF	57 (7.0%)	89 (12.1%)	0.57 (0.43–0.75)	0.61 (0.44–0.85)	0.004	

ACE-I, angiotensin-converting-enzyme inhibitors; ARB, angiotensin receptor blockers; HF, heart failure; HFmrEF, heart failure with mid-range ejection fraction; HFpEF, heart failure with preserved ejection fraction; HFrEF, heart failure with reduced ejection fraction; LVEF, left ventricular ejection fraction; HR, hazard ratio; CI, confidence interval.

In the sensitivity analysis using PS-matching, the results were consistent with those of the main analysis; in the matched cohorts from the three LVEF categories, the risks of the ACE-I/ARB group relative to the no ACE-I/ARB group tended to be lower in the HFrEF and HFmrEF groups (HFrEF, HR 0.79 [0.64–0.99], P = 0.037; HFmrEF, HR 0.73 [0.50–1.07], P = 0.10), but not in the HFpEF group (HFpEF, HR 0.98 [0.79–1.21], P = 0.86) (S4 Fig and S3–S5 Tables).

## Discussion

The main findings of the current study were as follows; first, in the current study population consisting of consecutive patients hospitalized for acute HF, patient characteristics were substantially different across the HFrEF, HFmrEF and HFpEF groups; second, ACE-I/ARB use as discharge medication relative to no ACE-I/ARB use was associated with significantly lower risk for the primary outcome measure (the composite of all-cause death and HF hospitalization) in HFrEF and HFmrEF, but not in HFpEF.

The differences in the patient characteristics across the three LVEF groups were consistent with those reported in the previous studies, whereas the age of patients in this registry was notably higher than that in previous studies. The GWTG-HF registry [7] showed that patients with HFmrEF were older (median 77 years old) and more likely to be women (49%) than HFrEF (median 72 years old, 37% women) and resembled HFpEF (median 78 years old, 65% women). Similarly in the current registry, HFmrEF patients were older and more likely to be female (median 80 years old, 40% women) compared to HFrEF (median 77 years old, 33% women) and intermediate between HFrEF and HFpEF (median 82 years old, 57% women). Furthermore, our registry was in line with the GWTG-HF registry in that the HFmrEF group were much more likely to have ischaemic etiology (41%) as the HFrEF group (45%), but in contrast to the HFpEF group (18%). On the other hand, the present registry reflected the aging society of Japan; this study included much older patients (median age 80) [8] than previous randomized clinical trials focusing on ACE-I/ARB [1, 2, 9, 10]. Thus, the higher incidence of 1-year outcomes in this study compared with those trials could be explained by the difference in the overall risk.

This study has indicated the possible effectiveness of ACE-I/ARB for improved long-term clinical outcomes even in patients with HFmrEF. The previous trials focusing on "HFpEF", defined as LVEF ≥45% or ≥40%, failed to show effectiveness of ACE-I/ARB to improve long-term clinical outcomes [9–11]. However, the recent reports have indicated the effectiveness of RAS inhibitors in HFmrEF as well as HFrEF [15–17]. For instance, the CHARM program [16] has recently reported that candesartan was effective in HFmrEF (HR 0.76 [95%CI 0.61–0.96], P = 0.02) to a similar degree as in HFrEF (HR 0.82 [95% CI 0.75–0.91], P<0.001), while that was not observed in HFpEF (HR 0.95 [95%CI 0.79–1.14], P = 0.57), although there was no significant interaction between LVEF category and candesartan treatment effect. The TOPCAT trial [15], which enrolled HF patients with LVEF ≥45%, indicated that the effect of spironolactone on the composite of cardiovascular death, heart failure hospitalization, or aborted cardiac arrest varied such that the greater benefit was observed in the lower LVEF spectrum. Furthermore, in the pooled analysis of PARADIGM-HF and PARAGON-HF [17], the effect of sacubitril/valsartan compared with a RAS inhibitor alone varied by LVEF with treatment benefits that appear to extend to HF patients with mildly reduced ejection fraction. The current study was consistent with these previous studies in that greater effectiveness of RAS inhibitors was suggested in the lower LVEF spectrum.

In this study population, prescription and uptitration of ACE-I/ARB were unsatisfactory even in the HFrEF group (S1 and S2 Figs). Although their prescription and uptitration for HFrEF are recommended in the guidelines [12], there would have been several reasons for the underprescription. The first reason would be that the study population was extremely advanced in age. The median age of the overall population was 80 and even that of the HFrEF group was 77. As age was clearly associated with prescription (S2 Fig), it would have been difficult to newly prescribe, continue and uptitrate ACE-I/ARB for elderly patients. Second, ACE-I/ARB could have been uptitrated for not all HF patients in the real clinical practice setting. For instance, blood pressure at presentation was associated with ACE-I/ARB as discharge medication (S1 and S2 Figs). In particular, the HFrEF group presented with lower blood pressure than the other two groups (Table 1). Further, renal function was also associated with prescription of ACE-I/ARB (S2 Fig). Given that nearly half of the HFrEF patients had estimated glomerular filtration rate <45 [ml/min/1.73m^2^] (Table 1), there would have been difficulty in ACE-I/ARB prescription and uptitration for HFrEF despite the guidelines' recommendation. Lastly, other RAS inhibitors such as mineralocorticoid receptor blockers were recommended for HFrEF patients as well as ACE-I/ARB, and they seemed to compete with ACE-I/ARB prescription and uptitration (S2 Fig). For these reasons, it might have been difficult to prescribe and uptitrate ACE-I/ARB for all the HFrEF patients. It should be acknowledged that we did not have detailed data on the reasons for non-use of ACE-I/ARB. Nevertheless, it can be said that this study clarified the cardiovascular drug prescription in the real clinical practice setting of an extremely aged Japanese society.

It should be noted that LVEF is one of the profiles of HF patients and that HF treatment should be considered according to not only the LVEF category, but also HF etiology. However, almost all previous trials of ACE-I/ARB for HF had enrollment criteria regarding LVEF, and it seems that LVEF modified the treatment effect [1–3, 9–11]. Treatment strategy for HFmrEF patients could be in line with that for HFrEF rather than HFpEF, because the mid-range LVEF (40–49%) cannot be regarded as normal spectrum as a lower boundary of normal LVEF range is 49–57% [24] and 52% in men and 54% in women [25]. In the impaired heart, activation of RAS is associated with myocardial remodeling. Increased levels of angiotensin II cause vasoconstriction, cellular hypertrophy, and interstitial fibrosis through the angiotensin II type 1 receptor [26]. It has been recently reported that increased plasma renin activity (PRA) is present in HFmrEF as well as HFrEF whereas HFpEF had low prevalence of PRA elevation [27]. ACE-I/ARB inhibit the RAS cascade and hence the benefit of the cardiovascular agents could be gained more in "reduced" ejection fraction. Although that benefit could be obtained in HFpEF as well, the higher risk of HFpEF than the other LVEF groups for non-cardiovascular death [28, 29] might compete with the beneficial effects of ACE-I/ARB. Furthermore, LVEF can change over time during the follow-up of HF patients [30], and the LVEF category tended to transition in HFrEF and HFmrEF patients, whereas it did not change so dynamically in HFpEF patients [31]. Given that HFmrEF has similar patient characteristics with HFrEF rather than HFpEF, this study might indicate that HFmrEF can be considered as a category whose prognosis is likely to be improved with ACE-I/ARB.

### Limitations

The current study has several limitations. First and most importantly, this study has an observational study design and thus there should have been residual confounding and indication bias to assess the effectiveness of ACE-I/ARB. The estimated effect of ACE-I/ARB for HFmrEF could be overinflated (HR 0.61 in this study versus HR 0.76 in the CHARM programme) [16]. Thus, the results of the current study are hypothesis-generating and should be interpreted with caution. However, specifically targeting the HFmrEF population in clinical trials is challenging and recent trials involving patients with HFmrEF had to be stopped due to difficulties in enrollment [32, 33]. The KCHF registry included all spectrum of LVEF and had comprehensive data on patient demographics, medical history, underlying heart disease, prehospital activities, socioeconomic status, signs, medications, laboratory tests, and echocardiography results, acute management in the emergency department, status at discharge, and clinical events during the index hospitalization. We performed extensive adjustment with most conceivable confounders that were considered to have clinical significance. Second, we could not assess prescription status and adherence during the follow-up and hence after the index hospitalization, patients in the ACE-I/ARB group might have stopped ACE-I/ARB and patients in the non-ACE-I/ARB group might have started ACE-I/ARB. Third, we did not have follow-up data of LVEF during the follow-up, although LVEF may have changed over time and thus some crossover from one group to another may have occurred [30]. Fourth, measuring LVEF measurement could have substantial inter-observer variability [34]. Fifth, the types and doses of ACE-I/ARB were heterogeneous in this population.

## Conclusions

ACE-I/ARB for patients who were hospitalized for acute HF was associated with significantly lower risk for a composite of all-cause death and HF hospitalization in HFrEF and HFmrEF, but not in HFpEF. ACE-I/ARB might be a potential treatment option in HFmrEF as in HFrEF.

## Supporting information

S1 File Definitions of baseline patient characteristics. (DOCX)

S1 Table Number of missing values. (DOCX)

S2 Table Types and doses of angiotensin-converting enzymes inhibitors and angiotensin receptor blockers. (DOCX)

S3 Table Patient characteristics in those with and without ACE-I/ARB, and propensity score-matching analysis in HFrEF. (DOCX)

S4 Table Patient characteristics in those with and without ACE-I/ARB, and propensity score-matching analysis in HFmrEF. (DOCX)

S5 Table Patient characteristics in those with and without ACE-I/ARB, and propensity score-matching analysis in HFpEF. (DOCX)

S6 Table Results of troponin measurement at admission. (DOCX)

S1 Fig Prescription and uptitration of ACE-I/ARB stratified by LVEF category. (DOCX)

S2 Fig Prescription of ACE-I/ARB and mineralocorticoid receptor blockers stratified by LVEF category. (DOCX)

S3 Fig Cumulative 1-year incidences of clinical outcomes in the three EF groups. (DOCX)

S4 Fig Propensity-score matching analysis and summarized results. (DOCX)
