# Supplementary material for: Impact of left ventricular ejection fraction on the effect of renin-angiotensin system blockers after an episode of acute heart failure: From the KCHF Registry
Source: PLoS One. 2020 Sep 14;15(9):e0239100. doi: 10.1371/journal.pone.0239100 (PMC7489562; doi:10.1371/journal.pone.0239100)
Supplement: S1 File — (DOCX) [file pone.0239100.s001.docx]

**S1 File: Definitions of baseline patient characteristics.**

The attending physicians or research assistants at each participating hospital collected comprehensive data on patient demographics, medical history, underlying heart disease, pre-hospital activities, socioeconomic status, signs, symptoms, medication, laboratory test, electrocardiogram, echocardiography, acute management during emergency room, status at discharge, and clinical events during the index hospitalization. The signs and symptoms were recorded at 4 time points: at hospital arrival; at admission; at 24 hours after hospital arrival (data not shown); and at discharge. Laboratory tests were performed at hospital presentation and at the nearest time to discharge. Left ventricular ejection fraction (LVEF) was measured at echocardiography departments, hospital wards, and emergency departments. We adopted the data in the earliest echocardiography as possible after admission. LVEF was measured by the biplane modified Simpson’s method.
