# Supplementary material for: Impact of left ventricular ejection fraction on the effect of renin-angiotensin system blockers after an episode of acute heart failure: From the KCHF Registry
Source: PLoS One. 2020 Sep 14;15(9):e0239100. doi: 10.1371/journal.pone.0239100 (PMC7489562; doi:10.1371/journal.pone.0239100)
Supplement: S1 Table — (DOCX) [file pone.0239100.s002.docx]

**S1 Table. Number of missing values.**

|  | Number of missing |
| --- | --- |
| Body mass index | 175 (4.7%) |
| Daily activities | 37 (1.0%) |
| Medical history |  |
| HF hospitalization | 67 (1.8%) |
| Current smoking | 67 (1.8%) |
| Activities of daily living | 37 (1.0%) |
| Vital signs at presentation |  |
| Blood pressure | 11 (0.3%) |
| Heart rate | 26 (0.7%) |
| NYHA class | 15 (0.4%) |
| Laboratory tests |  |
| BNP or NT-proBNP | 46 (1.2%) |
| Blood urea nitrogen | 11 (0.3%) |
| Creatinine | 7 (0.2%) |
| Albumin | 112 (3.0%) |
| Sodium | 11 (0.3%) |
| Potassium | 11 (0.3%) |
| Haemoglobin | 7 (0.2%) |

BNP, brain natriuretic peptide; NT-proBNP, N-terminal pro-B-type natriuretic peptide; NYHA, New York Heart Association.
