# Supplementary material for: Impact of left ventricular ejection fraction on the effect of renin-angiotensin system blockers after an episode of acute heart failure: From the KCHF Registry
Source: PLoS One. 2020 Sep 14;15(9):e0239100. doi: 10.1371/journal.pone.0239100 (PMC7489562; doi:10.1371/journal.pone.0239100)
Supplement: S2 Table — (DOCX) [file pone.0239100.s003.docx]

**S2 Table: Types and doses of angiotensin-converting enzymes inhibitors and angiotensin receptor blockers.**

**(A) Angiotensin-converting enzyme inhibitors.**

| Dose available  (N=886) |  |  | Standard dose |  | Total |
| --- | --- | --- | --- | --- | --- |
| Enalapril (mg) | dose <2.5 | 2.5≤ dose <5.0 | 5.0≤ dose <10 | 10≤ dose |  |
| N= | 59 | 299 | 218 | 39 | 615 |
| Lisinopril (mg) | dose <5.0 | 5.0≤ dose <10 | 10≤ dose <20 | 20≤ dose |  |
| N= | 0 | 1 | 9 | 5 | 15 |
| Imidapril (mg) | dose <2.5 | 2.5≤ dose <5.0 | 5.0≤ dose <10 | 10≤ dose |  |
| N= | 2 | 15 | 34 | 7 | 58 |
| Perindopril (mg) | dose <1.0 | 1.0≤ dose <2.0 | 2.0≤ dose <4.0 | 4.0≤ dose |  |
| N= | 0 | 8 | 70 | 114 | 192 |
| Temocapril (mg) | dose <1.0 | 1.0≤ dose <2.0 | 2.0≤ dose <4.0 | 4.0≤ dose |  |
| N= | 0 | 4 | 1 | 0 | 5 |
| Captopril (mg) | dose <18.75 | 18.75≤ dose <37.5 | 37.5≤ dose <75 | 75≤ dose |  |
| N= | 0 | 0 | 1 | 0 | 1 |

(B) Angiotensin receptor blockers.

| Dose available  (N=1224) |  |  | Standard dose |  | Total |
| --- | --- | --- | --- | --- | --- |
| Losartan (mg) | dose <25 | 25≤ dose <50 | 50≤ dose <100 | 100≤ dose |  |
| N= | 39 | 91 | 64 | 5 | 199 |
| Telmisartan (mg) | dose <20 | 20≤ dose <40 | 40≤ dose <80 | 80≤ dose |  |
| N= | 6 | 60 | 139 | 33 | 238 |
| Candesartan (mg) | dose <2.0 | 2.0≤ dose <4.0 | 4.0≤ dose <8.0 | 8.0≤ dose |  |
| N= | 1 | 15 | 82 | 92 | 190 |
| Valsartan (mg) | dose <20 | 20≤ dose <40 | 40≤ dose <80 | 80≤ dose |  |
| N= | 1 | 5 | 24 | 80 | 110 |
| Olmesartan (mg) | dose <10 | 10≤ dose <20 | 20≤ dose <40 | 40≤ dose |  |
| N= | 1 | 37 | 131 | 42 | 211 |
| Irbesartan (mg) | dose <25 | 25≤ dose <50 | 50≤ dose <100 | 100≤ dose |  |
| N= | 4 | 0 | 6 | 48 | 58 |
| Azilsartan (mg) | dose <10 | 10≤ dose <20 | 20≤ dose <40 | 40≤ dose |  |
| N= | 1 | 8 | 130 | 79 | 218 |

Standard doses were defined according to the drug information documents in Japan.
