# Supplementary material for: Impact of left ventricular ejection fraction on the effect of renin-angiotensin system blockers after an episode of acute heart failure: From the KCHF Registry
Source: PLoS One. 2020 Sep 14;15(9):e0239100. doi: 10.1371/journal.pone.0239100 (PMC7489562; doi:10.1371/journal.pone.0239100)
Supplement: S3 Table — (DOCX) [file pone.0239100.s004.docx]

**S3 Table: Baseline characteristics in the entire HFrEF cohort and in the propensity score-matched cohort: ACE-I/ARB versus no ACE-I/ARB.**

|  | Entire HFrEF cohort | | | |  | Propensity-score matched cohort | | | |  |
| --- | --- | --- | --- | --- | --- | --- | --- | --- | --- | --- |
|  | ACE-I/ARB | | No ACE-I/ARB | |  | ACE-I/ARB | | No ACE-I/ARB | |  |
|  | N=892 | | N=491 | | P value | N=372 | | N=372 | | SMD |
| Age [years] | 74 | [63–82] | 80 | [72–86] | <0.001 | 78 | [66–85] | 78 | [70–86] | 0.164 |
| Age ≥80* | 302 | (34%) | 247 | (50%) | <0.001 | 166 | (45%) | 170 | (46%) | 0.022 |
| Women* | 260 | (29%) | 198 | (40%) | <0.001 | 130 | (35%) | 136 | (37%) | 0.034 |
| BMI [kg/m2] | 23.4 | ±4.7 | 21.9 | ±4.3 | <0.001 | 22.9 | ±4.4 | 22.1 | ±4.5 | 0.164 |
| BMI <22 | 381 | (44%) | 247 | (52%) | 0.003 | 187 | (51%) | 177 | (50%) | 0.030 |
| Aetiology |  |  |  |  |  |  |  |  |  |  |
| Chronic CAD | 384 | (43%) | 236 | (48%) | 0.07 | 172 | (46%) | 180 | (48%) | 0.043 |
| Acute coronary syndrome | 56 | (6.3%) | 30 | (6.1%) | 0.90 | 34 | (9.1%) | 24 | (6.5%) | 0.100 |
| Hypertensive heart disease | 131 | (15%) | 53 | (11%) | 0.041 | 50 | (13%) | 42 | (11%) | 0.065 |
| Cardiomyopathy | 283 | (32%) | 114 | (23%) | 0.001 | 108 | (29%) | 92 | (25%) | 0.097 |
| Valvular heart disease | 70 | (7.8%) | 64 | (13%) | 0.002 | 33 | (8.9%) | 39 | (11%) | 0.055 |
| Medical history |  |  |  |  |  |  |  |  |  |  |
| HF hospitalization* | 319 | (37%) | 212 | (44%) | 0.007 | 153 | (42%) | 138 | (38%) | 0.084 |
| AF/AFL | 269 | (30%) | 169 | (34%) | 0.10 | 99 | (27%) | 120 | (32%) | 0.124 |
| Hypertension* | 605 | (68%) | 306 | (62%) | 0.04 | 223 | (60%) | 229 | (62%) | 0.033 |
| Diabetes mellitus* | 370 | (42%) | 197 | (40%) | 0.62 | 147 | (40%) | 153 | (41%) | 0.033 |
| Dyslipidemia | 380 | (43%) | 202 | (41%) | 0.60 | 152 | (41%) | 154 | (41%) | 0.011 |
| Prior myocardial infarction* | 270 | (30%) | 172 | (35%) | 0.07 | 131 | (35%) | 129 | (35%) | 0.011 |
| Prior stroke | 106 | (12%) | 84 | (17%) | 0.007 | 51 | (14%) | 61 | (16%) | 0.075 |
| Prior PCI/CABG | 268 | (30%) | 166 | (34%) | 0.15 | 119 | (32%) | 116 | (31%) | 0.017 |
| Current smoking | 171 | (19%) | 50 | (10%) | <0.001 | 57 | (16%) | 42 | (12%) | 0.116 |
| VT/VF | 74 | (8.3%) | 41 | (8.4%) | 0.97 | 30 | (8.1%) | 33 | (8.9%) | 0.029 |
| CRT | 42 | (4.7%) | 15 | (3.1%) | 0.14 | 16 | (4.3%) | 10 | (2.7%) | 0.088 |
| Lung disease | 105 | (12%) | 65 | (13%) | 0.43 | 45 | (12%) | 56 | (15%) | 0.086 |
| Cancer | 99 | (11%) | 81 | (17%) | 0.004 | 45 | (12%) | 62 | (17%) | 0.131 |
| Dementia | 97 | (11%) | 108 | (22%) | <0.001 | 53 | (14%) | 77 | (21%) | 0.171 |
| Social backgrounds |  |  |  |  |  |  |  |  |  |  |
| On job | 216 | (24%) | 55 | (11%) | <0.001 | 79 | (21%) | 51 | (14%) | 0.199 |
| Living alone | 215 | (24%) | 93 | (19%) | 0.03 | 77 | (21%) | 69 | (19%) | 0.054 |
| Activities of daily living |  |  |  |  |  |  |  |  |  |  |
| Ambulatory | 772 | (87%) | 372 | (77%) | <0.001 | 322 | (87%) | 289 | (79%) | 0.228 |
| Wheelchair | 83 | (9.4%) | 93 | (19%) |  | 40 | (11%) | 67 | (18%) |  |
| Bedridden | 29 | (3.3%) | 19 | (3.9%) |  | 7 | (1.9%) | 11 | (3.0%) |  |
| Vital signs at presentation |  |  |  |  |  |  |  |  |  |  |
| Systolic BP [mmHg] | 146.8 | ±33.4 | 135.9 | ±31.6 | <0.001 | 144.6 | ±32.5 | 138 | ±32.3 | 0.204 |
| Systolic BP <90* | 19 | (2.1%) | 22 | (4.5%) | 0.01 | 13 | (3.5%) | 10 | (2.7%) | 0.047 |
| Diastolic BP [mmHg] | 91.1 | ±23.9 | 83.4 | ±22.3 | <0.001 | 88.4 | ±24.2 | 85.5 | ±22.5 | 0.123 |
| Heart rate [/min] | 102.1 | ±24.9 | 98.9 | ±26.0 | 0.02 | 100.3 | ±22.5 | 101 | ±26.2 | 0.029 |
| Heart rate <60 | 15 | (1.7%) | 19 | (3.9%) | 0.01 | 7 | (1.9%) | 13 | (3.5%) | 0.100 |
| NYHA class III or IV* | 778 | (87%) | 435 | (90%) | 0.21 | 319 | (86%) | 328 | (89%) | 0.110 |
| LVEF [%] | 29.2 | ±7.0 | 28.8 | ±7.2 | 0.31 | 29.6 | ±7.0 | 28.6 | ±7.3 | 0.136 |
| Laboratory tests at admission |  |  |  |  |  |  |  |  |  |  |
| BNP [pg/ml] | 935 | [581–1582] | 1035 | [571–1711] | 0.18 | 970 | [609–1683] | 931 | [554–1652] | 0.027 |
| NT-proBNP [pg/ml] | 5401 | [2737–12449] | 8162 | [5294–19533] | 0.002 | 7784 | [3700–16395] | 7260 | [4839–14691] | 0.105 |
| BUN [mg/dl] | 22 | [17.0–30.5] | 27.8 | [19.1–43.7] | <0.001 | 24.3 | [18.2–34.0] | 25 | [18.5–38.3] | 0.103 |
| Creatinine [mg/dl] | 1.06 | [0.83–1.48] | 1.27 | [0.94–1.92] | <0.001 | 1.15 | [0.89–1.71] | 1.17 | [0.88–1.67] | 0.019 |
| Creatinine ≥2 | 105 | (12%) | 116 | (24%) | <0.001 | 67 | (18%) | 61 | (17%) | 0.038 |
| eGFR [ml/min/1.73m^2^] | 49.8 | [34.7–64.5] | 38.3 | [23.9–54.5] | <0.001 | 43.9 | [28.8–61.5] | 42.4 | [29.5–57.8] | 0.070 |
| eGFR <30* | 161 | (18%) | 176 | (36%) | <0.001 | 106 | (29%) | 96 | (26%) | 0.054 |
| Albumin [g/dl] | 3.6 | ±0.5 | 3.5 | ±0.5 | <0.001 | 3.5 | ±0.5 | 3.5 | ±0.5 | 0.036 |
| Albumin <3 | 85 | (9.7%) | 64 | (14%) | 0.03 | 43 | (12%) | 49 | (14%) | 0.061 |
| Sodium [mEq/l] | 139.3 | ±3.9 | 138.4 | ±4.6 | <0.001 | 139.2 | ±4.0 | 138.7 | ±4.4 | 0.117 |
| Sodium <135 | 86 | (9.7%) | 80 | (16%) | <0.001 | 40 | (11%) | 56 | (15%) | 0.133 |
| Potassium [mEq/l] | 4.15 | ±0.62 | 4.33 | ±0.76 | <0.001 | 4.22 | ±0.66 | 4.24 | ±0.70 | 0.032 |
| Potassium ≥5.0* | 77 | (8.7%) | 93 | (19%) | <0.001 | 49 | (13%) | 47 | (13%) | 0.012 |
| Haemoglobin [g/dl] | 12.6 | ±2.3 | 11.6 | ±2.4 | <0.001 | 12.2 | ±2.4 | 12 | ±2.4 | 0.102 |
| Anemia* | 442 | (50%) | 324 | (66%) | <0.001 | 216 | (58%) | 214 | (58%) | 0.005 |
| ACE-I/ARB at admission* | 492 | (55%) | 124 | (25%) | <0.001 | 142 | (38%) | 121 | (33%) | 0.118 |
| Medications at discharge |  |  |  |  |  |  |  |  |  |  |
| ACE-I | 481 | (54%) | 0 | (0.0%) | NA | 207 | (56%) | 0 | (0.0%) | NA |
| ARB | 418 | (47%) | 0 | (00%) | NA | 169 | (45%) | 0 | (0.0%) | NA |
| MRA* | 517 | (58%) | 205 | (42%) | <0.001 | 161 | (43%) | 186 | (50%) | 0.135 |
| β-blockers* | 755 | (85%) | 325 | (66%) | <0.001 | 275 | (74%) | 281 | (76%) | 0.037 |
| Loop diuretics* | 746 | (84%) | 400 | (82%) | 0.31 | 302 | (81%) | 304 | (82%) | 0.014 |
| Thiazide | 32 | (3.6%) | 31 | (6.3%) | 0.02 | 11 | (3.0%) | 28 | (7.5%) | 0.206 |
| Tolvaptan | 100 | (11%) | 69 | (14%) | 0.12 | 49 | (13%) | 49 | (13%) | <0.001 |
| Digoxin | 55 | (6.2%) | 33 | (6.7%) | 0.69 | 14 | (3.8%) | 29 | (7.8%) | 0.173 |
| Warfarin | 210 | (24%) | 119 | (24%) | 0.77 | 87 | (23%) | 87 | (23%) | <0.001 |
| DOAC | 153 | (17%) | 74 | (15%) | 0.32 | 48 | (13%) | 69 | (19%) | 0.156 |

*Variables relevant to the choice of ACE-I/ARB for logistic regression model to develop a propensity score.

ACE-I, angiotensin-converting-enzyme inhibitors; AF, atrial fibrillation; AFL, atrial flutter; ARB, angiotensin receptor blockers; BMI, body mass index; BNP, brain natriuretic peptide; BP, blood pressure; BUN, blood urea nitrogen; CABG, coronary artery bypass grafting; CAD, coronary artery disease; DOAC, direct oral anticoagulants; eGFR, estimated glomerular filtration rate; HF, heart failure; HFmrEF, heart failure with mid-range ejection fraction; HFpEF, heart failure with preserved ejection fraction; HFrEF, heart failure with reduced ejection fraction; LVEF, left ventricular ejection fraction; MRA, mineralocorticoid receptor antagonists; NT-proBNP, N-terminal pro-B-type natriuretic peptide; NYHA, New York Heart Association; PCI, percutaneous coronary intervention; SMD, standard mean difference; VF, ventricular fibrillation; VT, ventricular tachycardia.
