# Supplementary material for: Impact of left ventricular ejection fraction on the effect of renin-angiotensin system blockers after an episode of acute heart failure: From the KCHF Registry
Source: PLoS One. 2020 Sep 14;15(9):e0239100. doi: 10.1371/journal.pone.0239100 (PMC7489562; doi:10.1371/journal.pone.0239100)
Supplement: S4 Table — (DOCX) [file pone.0239100.s005.docx]

**S4 Table: Baseline characteristics in the entire HFmrEF cohort and in the propensity score-matched cohort: ACE-I/ARB versus no ACE-I/ARB.**

|  | Entire HFmrEF cohort | | | |  | Propensity-score matched cohort | | | |  |
| --- | --- | --- | --- | --- | --- | --- | --- | --- | --- | --- |
|  | ACE-I/ARB | | No ACE-I/ARB | |  | ACE-I/ARB | | No ACE-I/ARB | |  |
|  | N=400 | | N=303 | | P value | N=154 | | N=154 | | SMD |
| Age [years] | 79 | [72–85] | 82 | [72–87] | 0.03 | 80 | [71–86] | 81 | [72–86] | 0.131 |
| Age ≥80* | 195 | (49%) | 171 | (56%) | 0.043 | 78 | (51%) | 81 | (53%) | 0.039 |
| Women* | 153 | (38%) | 130 | (43%) | 0.21 | 73 | (47%) | 63 | (41%) | 0.131 |
| BMI [kg/m2] | 23.1 | ±4.5 | 22.1 | ±3.9 | 0.004 | 22.6 | ±4.4 | 22.6 | ±4.0 | 0.004 |
| BMI <22 | 165 | (43%) | 145 | (51%) | 0.043 | 69 | (47%) | 64 | (45%) | 0.031 |
| Aetiology |  |  |  |  |  |  |  |  |  |  |
| Choronic CAD | 166 | (42%) | 123 | (41%) | 0.81 | 48 | (31%) | 60 | (39%) | 0.164 |
| Acute coronary syndrome | 36 | (9.0%) | 19 | (6.3%) | 0.18 | 13 | (8.4%) | 10 | (6.5%) | 0.074 |
| Hypertensive heart disease | 115 | (29%) | 64 | (21%) | 0.02 | 53 | (34%) | 33 | (21%) | 0.293 |
| Cardiomyopathy | 36 | (9.0%) | 35 | (12%) | 0.27 | 16 | (10%) | 18 | (12%) | 0.041 |
| Valvular | 70 | (18%) | 63 | (21%) | 0.27 | 33 | (21%) | 32 | (21%) | 0.016 |
| Medical history |  |  |  |  |  |  |  |  |  |  |
| Prior HF hospitalization* | 127 | (32%) | 110 | (37%) | 0.19 | 46 | (31%) | 46 | (31%) | <0.001 |
| AF/AFL | 155 | (39%) | 137 | (45%) | 0.09 | 53 | (34%) | 75 | (49%) | 0.293 |
| Hypertension* | 324 | (81%) | 212 | (70%) | 0.001 | 121 | (79%) | 118 | (77%) | 0.047 |
| Diabetes mellitus* | 156 | (39%) | 130 | (43%) | 0.30 | 51 | (33%) | 50 | (33%) | 0.014 |
| Dyslipidemia | 173 | (43%) | 120 | (40%) | 0.33 | 56 | (36%) | 54 | (35%) | 0.027 |
| Prior myocardial infarction* | 128 | (32%) | 88 | (29%) | 0.40 | 32 | (21%) | 42 | (27%) | 0.152 |
| Prior stroke | 70 | (18%) | 46 | (15%) | 0.41 | 28 | (18%) | 19 | (12%) | 0.163 |
| Prior PCI/CABG | 129 | (32%) | 92 | (30%) | 0.59 | 40 | (26%) | 42 | (27%) | 0.029 |
| Current smoking | 66 | (17%) | 35 | (12%) | 0.08 | 29 | (19%) | 18 | (12%) | 0.187 |
| VT/VF | 10 | (2.5%) | 8 | (2.6%) | 0.91 | 3 | (1.9%) | 2 | (1.3%) | 0.051 |
| CRT | 5 | (1.2%) | 4 | (1.3%) | 0.94 | 2 | (1.3%) | 3 | (1.9%) | 0.051 |
| Lung disease | 41 | (10%) | 34 | (11%) | 0.68 | 18 | (12%) | 18 | (12%) | <0.001 |
| Cancer | 52 | (13%) | 52 | (17%) | 0.12 | 18 | (12%) | 23 | (15%) | 0.096 |
| Dementia | 60 | (15%) | 59 | (20%) | 0.12 | 23 | (15%) | 33 | (21%) | 0.169 |
| Social backgrounds |  |  |  |  |  |  |  |  |  |  |
| On job | 61 | (15%) | 30 | (9.9%) | 0.036 | 20 | (13%) | 19 | (12%) | 0.020 |
| Living alone | 86 | (22%) | 64 | (21%) | 0.90 | 34 | (22%) | 30 | (20%) | 0.064 |
| Activities of daily living |  |  |  |  |  |  |  |  |  |  |
| Ambulatory | 341 | (86%) | 229 | (76%) | 0.002 | 132 | (87%) | 121 | (80%) | 0.099 |
| Wheelchair | 51 | (13%) | 59 | (20%) |  | 19 | (13%) | 24 | (16%) |  |
| Bedridden | 5 | (1.3%) | 12 | (4.0%) |  | 1 | (0.7%) | 6 | (4.0%) |  |
| Vital signs at presentation |  |  |  |  |  |  |  |  |  |  |
| Systolic BP [mmHg] | 158.5 | ±36.3 | 146.3 | ±33.7 | <0.001 | 161.3 | ±40.1 | 146.3 | ±33.2 | 0.407 |
| Systolic BP <90* | 6 | (1.5%) | 6 | (2.0%) | 0.63 | 3 | (1.9%) | 4 | (2.6%) | 0.044 |
| Diastolic BP [mmHg] | 90.4 | ±24.6 | 83.6 | ±22.4 | <0.001 | 91.1 | ±27.2 | 85.6 | ±20.9 | 0.227 |
| Heart rate [/min] | 99.1 | ±28.4 | 99.6 | ±28.2 | 0.82 | 98.8 | ±30.2 | 100.8 | ±28.3 | 0.071 |
| Heart rate <60 | 23 | (5.8%) | 14 | (4.7%) | 0.52 | 11 | (7.2%) | 5 | (3.3%) | 0.176 |
| NYHA class III or IV* | 351 | (88%) | 255 | (84%) | 0.15 | 135 | (89%) | 137 | (89%) | 0.005 |
| LVEF [%] | 44.3 | ±2.9 | 44.2 | ±2.9 | 0.70 | 44.5 | ±3.0 | 44.5 | ±3.0 | 0.003 |
| Laboratory tests at admission |  |  |  |  |  |  |  |  |  |  |
| BNP [pg/ml] | 746 | [431–1264] | 867 | [460–1294] | 0.21 | 809 | [469–1374] | 776 | [451–1194] | 0.181 |
| NT-proBNP [pg/ml] | 6281 | [2682–17556] | 6494 | [3382–20340] | 0.66 | 7584 | [4431–16376] | 5527 | [2365–11590] | 0.005 |
| BUN [mg/dl] | 22.1 | [17.0–30.6] | 27 | [19.0–39.5] | <0.001 | 24.0 | [17.0–32.8] | 25.3 | [18.0–36.0] | 0.070 |
| Creatinine [mg/dl] | 1.05 | [0.80–1.50] | 1.25 | [0.85–2.05] | <0.001 | 1.04 | [0.75–1.77] | 1.13 | [0.84–1.80] | 0.013 |
| Creatinine ≥2 | 58 | (15%) | 80 | (26%) | <0.001 | 30 | (20%) | 32 | (21%) | 0.032 |
| eGFR [ml/min/1.73m^2^] | 48.4 | [33.2–63.4] | 37.5 | [22.3–59.5] | <0.001 | 45.6 | [26.5–63.6] | 41.5 | [26.3–61.9] | 0.115 |
| eGFR <30* | 86 | (22%) | 118 | (39%) | <0.001 | 47 | (31%) | 46 | (30%) | 0.014 |
| Albumin [g/dl] | 3.5 | ±0.5 | 3.4 | ±0.5 | <0.001 | 3.5 | ±0.4 | 3.4 | ±0.5 | 0.154 |
| Albumin <3 | 44 | (11%) | 57 | (19%) | 0.003 | 18 | (12%) | 27 | (18%) | 0.166 |
| Sodium [mEq/l] | 139.4 | ±4.4 | 138.9 | ±4.1 | 0.11 | 139.3 | ±4.5 | 139.1 | ±4.0 | 0.058 |
| Sodium <135 | 38 | (9.5%) | 36 | (12%) | 0.31 | 16 | (10%) | 20 | (13%) | 0.083 |
| Potassium [mEq/l] | 4.15 | ±0.61 | 4.25 | ±0.68 | 0.037 | 4.16 | ±0.64 | 4.23 | ±0.66 | 0.12 |
| Potassium ≥5.0* | 38 | (9.5%) | 39 | (13%) | 0.16 | 16 | (10%) | 19 | (12%) | 0.064 |
| Haemoglobin [g/dl] | 11.9 | ±2.3 | 11.0 | ±2.2 | <0.001 | 11.7 | ±2.2 | 11.8 | ±2.2 | 0.051 |
| Anemia* | 241 | (60%) | 230 | (76%) | <0.001 | 103 | (67%) | 97 | (63%) | 0.082 |
| ACE-I/ARB at admission* | 242 | (61%) | 65 | (22%) | <0.001 | 64 | (42%) | 62 | (40%) | 0.026 |
| Medications at discharge |  |  |  |  |  |  |  |  |  |  |
| ACE-I | 158 | (40%) | 0 | (0.0%) | NA | 65 | (42%) | 0 | (0.0%) | NA |
| ARB | 243 | (61%) | 0 | (0.0%) | NA | 89 | (58%) | 0 | (0.0%) | NA |
| MRA* | 187 | (47%) | 123 | (41%) | 0.10 | 64 | (42%) | 68 | (44%) | 0.053 |
| β-blockers* | 310 | (78%) | 194 | (64%) | <0.001 | 110 | (71%) | 110 | (71%) | <0.001 |
| Loop diuretics* | 320 | (80%) | 240 | (79%) | 0.80 | 126 | (82%) | 122 | (79%) | 0.066 |
| Thiazide | 19 | (4.8%) | 17 | (5.6%) | 0.61 | 8 | (5.2%) | 5 | (3.2%) | 0.097 |
| Tolvaptan | 31 | (7.8%) | 25 | (8.3%) | 0.81 | 14 | (9.1%) | 11 | (7.1%) | 0.071 |
| Digoxin | 14 | (3.5%) | 16 | (5.3%) | 0.25 | 5 | (3.2%) | 7 | (4.5%) | 0.067 |
| Warfarin | 69 | (17%) | 86 | (28%) | <0.001 | 23 | (15%) | 46 | (30%) | 0.364 |
| DOAC | 93 | (23%) | 60 | (20%) | 0.27 | 38 | (25%) | 35 | (23%) | 0.046 |

*Variables relevant to the choice of ACE-I/ARB for logistic regression model to develop a propensity score.

ACE-I, angiotensin-converting-enzyme inhibitors; AF, atrial fibrillation; AFL, atrial flutter; ARB, angiotensin receptor blockers; BMI, body mass index; BNP, brain natriuretic peptide; BP, blood pressure; BUN, blood urea nitrogen; CABG, coronary artery bypass grafting; CAD, coronary artery disease; DOAC, direct oral anticoagulants; eGFR, estimated glomerular filtration rate; HF, heart failure; HFmrEF, heart failure with mid-range ejection fraction; HFpEF, heart failure with preserved ejection fraction; HFrEF, heart failure with reduced ejection fraction; LVEF, left ventricular ejection fraction; MRA, mineralocorticoid receptor antagonists; NT-proBNP, N-terminal pro-B-type natriuretic peptide; NYHA, New York Heart Association; PCI, percutaneous coronary intervention; SMD, standard mean difference; VF, ventricular fibrillation; VT, ventricular tachycardia.
