# Supplementary material for: Impact of left ventricular ejection fraction on the effect of renin-angiotensin system blockers after an episode of acute heart failure: From the KCHF Registry
Source: PLoS One. 2020 Sep 14;15(9):e0239100. doi: 10.1371/journal.pone.0239100 (PMC7489562; doi:10.1371/journal.pone.0239100)
Supplement: S5 Table — (DOCX) [file pone.0239100.s006.docx]

**S5 Table: Baseline characteristics in the entire HFpEF cohort and in the propensity score-matched cohort: ACE-I/ARB versus no ACE-I/ARB.**

|  | Entire HFpEF cohort | | | |  | Propensity-score matched cohort | | | |  |
| --- | --- | --- | --- | --- | --- | --- | --- | --- | --- | --- |
|  | ACE-I/ARB | | No ACE-I/ARB | |  | ACE-I/ARB | | No ACE-I/ARB | |  |
|  | N=846 | | N=785 | | P value | N=426 | | N=426 | | SMD |
| Age [years] | 82 | [75–87] | 83 | [76–88] | 0.002 | 82 | [75–87] | 83 | [76–88] | 0.087 |
| Age ≥80* | 502 | (59%) | 511 | (65%) | 0.02 | 257 | (60%) | 267 | (63%) | 0.048 |
| Women* | 479 | (57%) | 448 | (57%) | 0.85 | 229 | (54%) | 243 | (57%) | 0.066 |
| BMI [kg/m2] | 23.4 | ±4.7 | 22.5 | ±4.1 | <0.001 | 23.1 | ±4.5 | 23.2 | ±4.2 | 0.019 |
| BMI <22 | 346 | (43%) | 354 | (49%) | 0.02 | 186 | (46%) | 171 | (43%) | 0.055 |
| Aetiology |  |  |  |  |  |  |  |  |  |  |
| Chronic CAD | 170 | (20%) | 126 | (16%) | 0.03 | 72 | (17%) | 73 | (17%) | 0.006 |
| Acute coronary syndrome | 39 | (4.6%) | 25 | (3.2%) | 0.14 | 23 | (5.4%) | 15 | (3.5%) | 0.091 |
| Hypertensive heart disease | 343 | (41%) | 221 | (28%) | <0.001 | 178 | (42%) | 144 | (34%) | 0.165 |
| Cardiomyopathy | 41 | (4.8%) | 47 | (6.0%) | 0.31 | 18 | (4.2%) | 21 | (4.9%) | 0.034 |
| Valvular | 214 | (25%) | 254 | (32%) | 0.002 | 111 | (26%) | 133 | (31%) | 0.114 |
| Medical history |  |  |  |  |  |  |  |  |  |  |
| Prior HF hospitalization* | 277 | (33%) | 272 | (36%) | 0.29 | 118 | (28%) | 122 | (29%) | 0.032 |
| AF/AFL | 403 | (48%) | 417 | (53%) | 0.03 | 193 | (45%) | 225 | (53%) | 0.151 |
| Hypertension* | 730 | (86%) | 513 | (65%) | <0.001 | 343 | (81%) | 346 | (81%) | 0.018 |
| Diabetes mellitus* | 321 | (38%) | 218 | (28%) | <0.001 | 131 | (31%) | 136 | (32%) | 0.025 |
| Dyslipidemia | 333 | (39%) | 244 | (31%) | <0.001 | 142 | (33%) | 155 | (36%) | 0.064 |
| Prior myocardial infarction* | 105 | (12%) | 73 | (9.3%) | 0.044 | 44 | (10%) | 38 | (8.9%) | 0.048 |
| Prior stroke | 145 | (17%) | 139 | (18%) | 0.76 | 72 | (17%) | 75 | (18%) | 0.019 |
| Prior PCI/CABG | 181 | (21%) | 117 | (15%) | 0.001 | 71 | (17%) | 68 | (16%) | 0.019 |
| Current smoking | 79 | (9.5%) | 51 | (6.6%) | 0.03 | 43 | (10%) | 27 | (6.4%) | 0.143 |
| VT/VF | 8 | (0.9%) | 13 | (1.7%) | 0.20 | 4 | (0.9%) | 6 | (1.4%) | 0.044 |
| CRT | 3 | (0.4%) | 2 | (0.3%) | 0.72 | 3 | (0.7%) | 1 | (0.2%) | 0.069 |
| Lung disease | 130 | (15%) | 113 | (14%) | 0.58 | 63 | (15%) | 53 | (12%) | 0.068 |
| Cancer | 144 | (17%) | 107 | (14%) | 0.058 | 91 | (21%) | 61 | (14%) | 0.185 |
| Dementia | 164 | (19%) | 167 | (21%) | 0.34 | 94 | (22%) | 79 | (19%) | 0.088 |
| Social backgrounds |  |  |  |  |  |  |  |  |  |  |
| On job | 77 | (9.1%) | 55 | (7.0%) | 0.12 | 49 | (12%) | 29 | (6.8%) | 0.163 |
| Living alone | 188 | (22%) | 148 | (19%) | 0.09 | 84 | (20%) | 94 | (22%) | 0.058 |
| Activities of daily living |  |  |  |  |  |  |  |  |  |  |
| Ambulatory | 664 | (79%) | 563 | (73%) | 0.001 | 333 | (79%) | 325 | (77%) | 0.144 |
| Wheelchair | 156 | (19%) | 168 | (22%) |  | 78 | (19%) | 76 | (18%) |  |
| Bedridden | 20 | (2.4%) | 44 | (5.7%) |  | 11 | (2.6%) | 23 | (5.4%) |  |
| Vital signs at presentation |  |  |  |  |  |  |  |  |  |  |
| Systolic BP [mmHg] | 158.5 | ±34.6 | 142.5 | ±34.9 | <0.001 | 159.9 | ±35.5 | 146.7 | ±34.0 | 0.380 |
| Systolic BP <90* | 7 | (0.8%) | 35 | (4.5%) | <0.001 | 6 | (1.4%) | 7 | (1.7%) | 0.020 |
| Diastolic BP [mmHg] | 83.8 | ±24.1 | 78.7 | ±22.9 | <0.001 | 86.5 | ±24.4 | 80.5 | ±22.5 | 0.253 |
| Heart rate [/min] | 89.4 | ±28.3 | 91.1 | ±28.2 | 0.22 | 91.2 | ±29.5 | 91.7 | ±27.3 | 0.020 |
| Heart rate <60 | 92 | (11%) | 87 | (11%) | 0.86 | 44 | (10%) | 44 | (11%) | 0.003 |
| NYHA class III or IV* | 734 | (87%) | 669 | (86%) | 0.48 | 372 | (88%) | 375 | (88%) | 0.022 |
| LVEF [%] | 62.3 | ±7.7 | 61.5 | ±7.2 | 0.056 | 62.4 | ±7.9 | 61.7 | ±7.2 | 0.092 |
| Laboratory tests at admission |  |  |  |  |  |  |  |  |  |  |
| BNP [pg/ml] | 467 | [282–819] | 525 | [281–916] | 0.10 | 451 | [264–765] | 511 | [282–880] | 0.152 |
| NT-proBNP [pg/ml] | 4650 | [2095–8878] | 4865 | [2475–10664] | 0.69 | 5316 | [1983–9998] | 4386 | [2520–10283] | 0.003 |
| BUN [mg/dl] | 22.1 | [17.0–31.5] | 25.0 | [18.0–36.8] | <0.001 | 22.0 | [16.3–32.7] | 23.4 | [17.8–32.6] | 0.063 |
| Creatinine [mg/dl] | 1.02 | [0.79–1.43] | 1.13 | [0.82–1.68] | 0.001 | 0.99 | [0.75–1.42] | 1.06 | [0.81–1.48] | 0.064 |
| Creatinine ≥2 | 98 | (12%) | 141 | (18%) | <0.001 | 51 | (12%) | 56 | (13%) | 0.035 |
| eGFR [ml/min/1.73m^2^] | 45.6 | [31.4–60.1] | 41.2 | [26.8–58.8] | <0.001 | 47.1 | [31.5–65.8] | 42.9 | [30.6–59.1] | 0.163 |
| eGFR <30* | 187 | (22%) | 250 | (32%) | <0.001 | 98 | (23%) | 103 | (24%) | 0.026 |
| Albumin [g/dl] | 3.5 | ±0.5 | 3.4 | ±0.5 | 0.001 | 3.5 | ±0.5 | 3.5 | ±0.5 | 0.021 |
| Albumin <3 | 108 | (13%) | 122 | (16%) | 0.11 | 70 | (17%) | 50 | (12%) | 0.137 |
| Sodium [mEq/l] | 139.6 | ±4.1 | 139.0 | ±4.2 | 0.005 | 139.4 | ±4.2 | 139.4 | ±4.0 | 0.001 |
| Sodium <135 | 87 | (10%) | 106 | (14%) | 0.046 | 52 | (12%) | 43 | (10%) | 0.067 |
| Potassium [mEq/l] | 4.15 | ±0.64 | 4.24 | ±0.71 | 0.008 | 4.13 | ±0.68 | 4.19 | ±0.69 | 0.091 |
| Potassium ≥5.0* | 82 | (9.7%) | 103 | (13%) | 0.03 | 41 | (9.6%) | 45 | (11%) | 0.031 |
| Haemoglobin [g/dl] | 11.1 | ±2.2 | 10.8 | ±2.1 | 0.03 | 11.2 | ±2.3 | 11 | ±2.2 | 0.109 |
| Anemia* | 622 | (74%) | 598 | (76%) | 0.23 | 300 | (71%) | 308 | (72%) | 0.038 |
| ACE-I/ARB at admission* | 584 | (69%) | 191 | (24%) | <0.001 | 182 | (43%) | 186 | (44%) | 0.019 |
| Medications at discharge |  |  |  |  |  |  |  |  |  |  |
| ACE-I | 270 | (32%) | 0 | (0.0%) | NA | 177 | (42%) | 0 | (0.0%) | NA |
| ARB | 585 | (69%) | 0 | (0.0%) | NA | 253 | (59%) | 0 | (0.0%) | NA |
| MRA* | 347 | (41%) | 299 | (38%) | 0.23 | 160 | (38%) | 172 | (40%) | 0.058 |
| β-blockers* | 496 | (59%) | 389 | (50%) | <0.001 | 224 | (53%) | 220 | (52%) | 0.019 |
| Loop diuretics* | 691 | (82%) | 618 | (79%) | 0.13 | 339 | (80%) | 347 | (82%) | 0.047 |
| Thiazide | 62 | (7.3%) | 57 | (7.3%) | 0.96 | 27 | (6.3%) | 28 | (6.6%) | 0.010 |
| Tolvaptan | 64 | (7.6%) | 101 | (13%) | <0.001 | 34 | (8.0%) | 55 | (13%) | 0.162 |
| Digoxin | 45 | (5.3%) | 48 | (6.1%) | 0.49 | 26 | (6.1%) | 30 | (7.0%) | 0.038 |
| Warfarin | 187 | (22%) | 253 | (32%) | <0.001 | 72 | (17%) | 132 | (31%) | 0.335 |
| DOAC | 217 | (26%) | 163 | (21%) | 0.02 | 127 | (30%) | 90 | (21%) | 0.200 |

*Variables relevant to the choice of ACE-I/ARB for logistic regression model to develop a propensity score.

ACE-I, angiotensin-converting-enzyme inhibitors; AF, atrial fibrillation; AFL, atrial flutter; ARB, angiotensin receptor blockers; BMI, body mass index; BNP, brain natriuretic peptide; BP, blood pressure; BUN, blood urea nitrogen; CABG, coronary artery bypass grafting; CAD, coronary artery disease; DOAC, direct oral anticoagulants; eGFR, estimated glomerular filtration rate; HF, heart failure; HFmrEF, heart failure with mid-range ejection fraction; HFpEF, heart failure with preserved ejection fraction; HFrEF, heart failure with reduced ejection fraction; LVEF, left ventricular ejection fraction; MRA, mineralocorticoid receptor antagonists; NT-proBNP, N-terminal pro-B-type natriuretic peptide; NYHA, New York Heart Association; PCI, percutaneous coronary intervention; SMD, standard mean difference; VF, ventricular fibrillation; VT, ventricular tachycardia.
