# Supplementary material for: Impact of left ventricular ejection fraction on the effect of renin-angiotensin system blockers after an episode of acute heart failure: From the KCHF Registry
Source: PLoS One. 2020 Sep 14;15(9):e0239100. doi: 10.1371/journal.pone.0239100 (PMC7489562; doi:10.1371/journal.pone.0239100)
Supplement: S6 Table — (DOCX) [file pone.0239100.s007.docx]

**S6 Table: Results of troponin measurement at admission.**

A) HFrEF versus HFmrEF versus HFpEF.

|  | HFrEF | HFmrEF | HFpEF | P value | N |
| --- | --- | --- | --- | --- | --- |
| Troponin T | 0.05 [0.03–0.11] | 0.05 [0.03–0.14] | 0.04 [0.02–0.08] | <0.001 | 935 |
| Troponin I | 0.07 [0.04–0.24] | 0.07 [0.02–0.23] | 0.04 [0.02–0.13] | <0.001 | 1552 |

B) ACE-I/ARB versus no ACE-I/ARB; stratified by LVEF category.

|  | ACE-I/ARB | No ACE-I/ARB |  |  |
| --- | --- | --- | --- | --- |
| HFrEF |  |  | P value | N |
| Troponin T | 0.05 [0.03–0.10] | 0.06 [0.03–0.14] | 0.12 | 325 |
| Troponin I | 0.07 [0.03–0.23] | 0.08 [0.04–0.28] | 0.11 | 635 |
| HFmrEF |  |  |  |  |
| Troponin T | 0.05 [0.03–0.12] | 0.05 [0.03–0.17] | 0.94 | 177 |
| Troponin I | 0.06 [0.02–0.25] | 0.07 [0.02–0.25] | 0.82 | 299 |
| HFpEF |  |  |  |  |
| Troponin T | 0.04 [0.02–0.08] | 0.04 [0.02–0.08] | 0.49 | 433 |
| Troponin I | 0.04 [0.02–0.14] | 0.05 [0.02–0.12] | 0.71 | 618 |

HFmrEF, heart failure with mid-range ejection fraction; HFpEF, heart failure with preserved ejection fraction; HFrEF, heart failure with reduced ejection fraction; LVEF, left ventricular ejection fraction.
