# Supplementary material for: Impact of left ventricular ejection fraction on the effect of renin-angiotensin system blockers after an episode of acute heart failure: From the KCHF Registry
Source: PLoS One. 2020 Sep 14;15(9):e0239100. doi: 10.1371/journal.pone.0239100 (PMC7489562; doi:10.1371/journal.pone.0239100)
Supplement: S1 Fig — (DOCX) [file pone.0239100.s008.docx]

**S1 Fig: Prescription and uptitration of ACE-I/ARB stratified by LVEF category.**

**A) Overall population; stratified by LVEF category.**

**
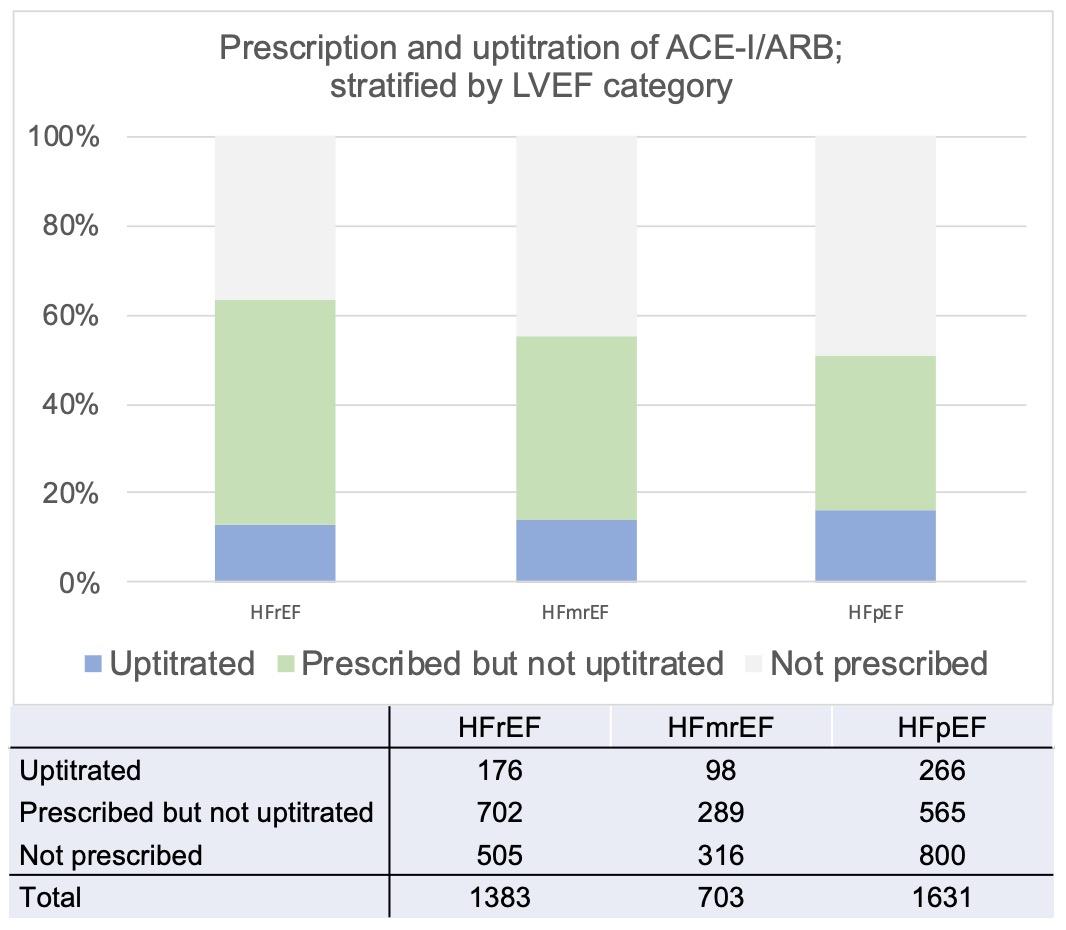
**

**B) HFrEF group; stratified by systolic BP at presentation.**

**
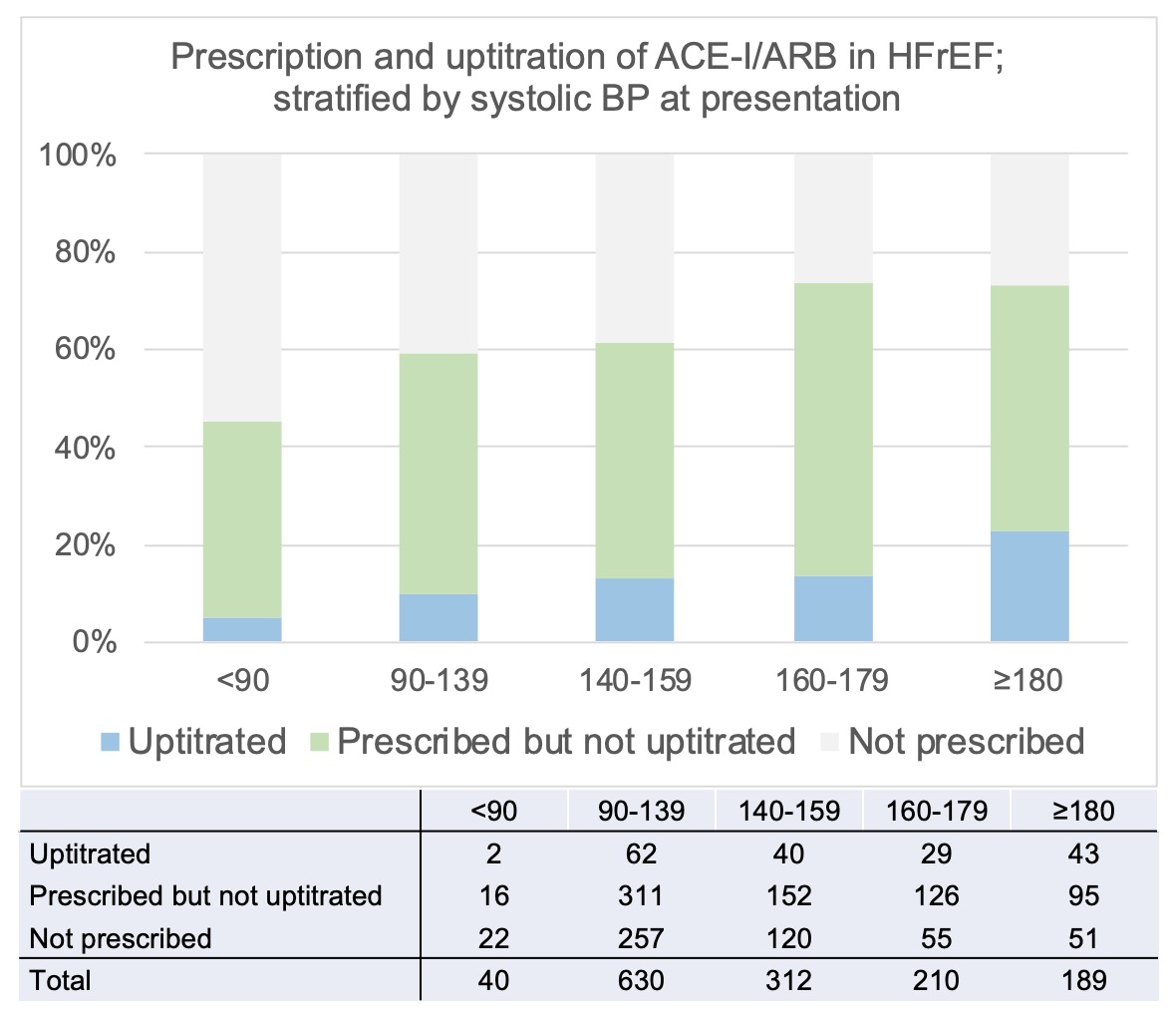
**

**C) HFmrEF group; stratified by systolic BP at presentation.**

**
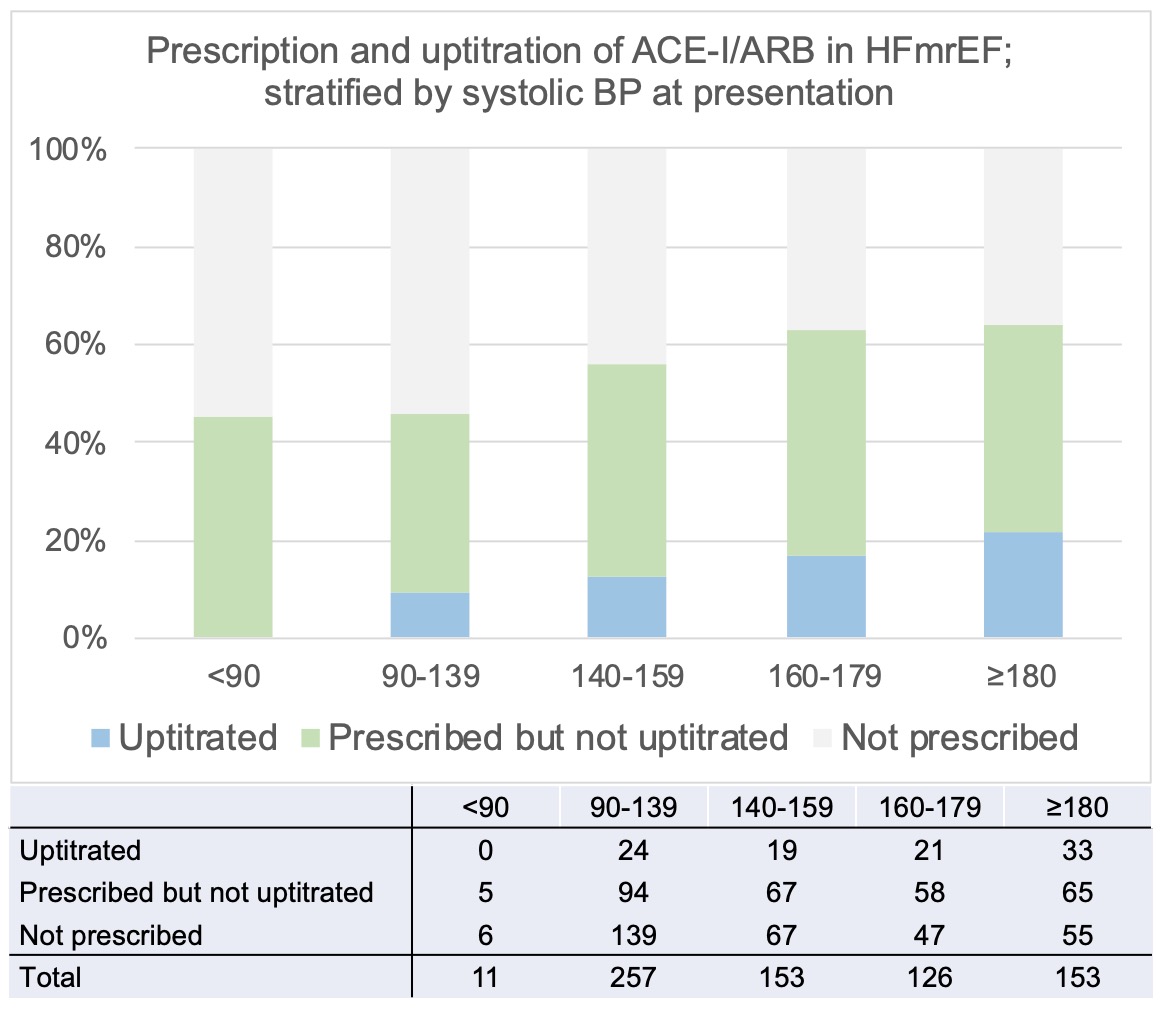
**

**D) HFpEF group; stratified by systolic BP at presentation.**

**
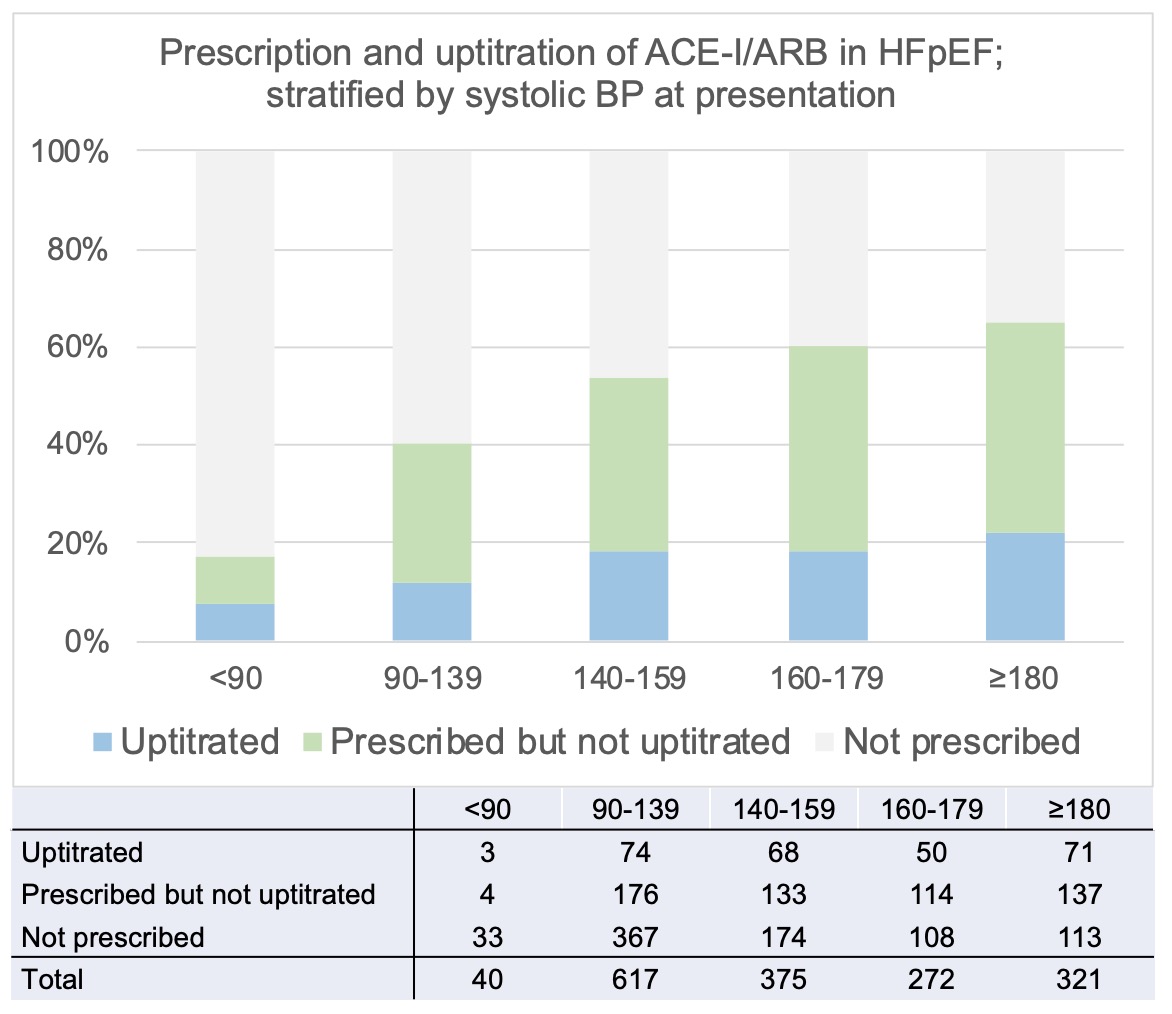
**

ACE-I, angiotensin-converting-enzyme inhibitors; ARB, angiotensin receptor blockers BP, blood pressure; HFmrEF, heart failure with mid-range ejection fraction; HFpEF, heart failure with preserved ejection fraction; HFrEF, heart failure with reduced ejection fraction; LVEF, left ventricular ejection fraction.
