# Supplementary material for: Impact of left ventricular ejection fraction on the effect of renin-angiotensin system blockers after an episode of acute heart failure: From the KCHF Registry
Source: PLoS One. 2020 Sep 14;15(9):e0239100. doi: 10.1371/journal.pone.0239100 (PMC7489562; doi:10.1371/journal.pone.0239100)
Supplement: S2 Fig — (DOCX) [file pone.0239100.s009.docx]

**S2 Fig: Prescription of ACE-I/ARB and mineralocorticoid receptor blockers stratified by LVEF category.**

**A) Stratified by age.**


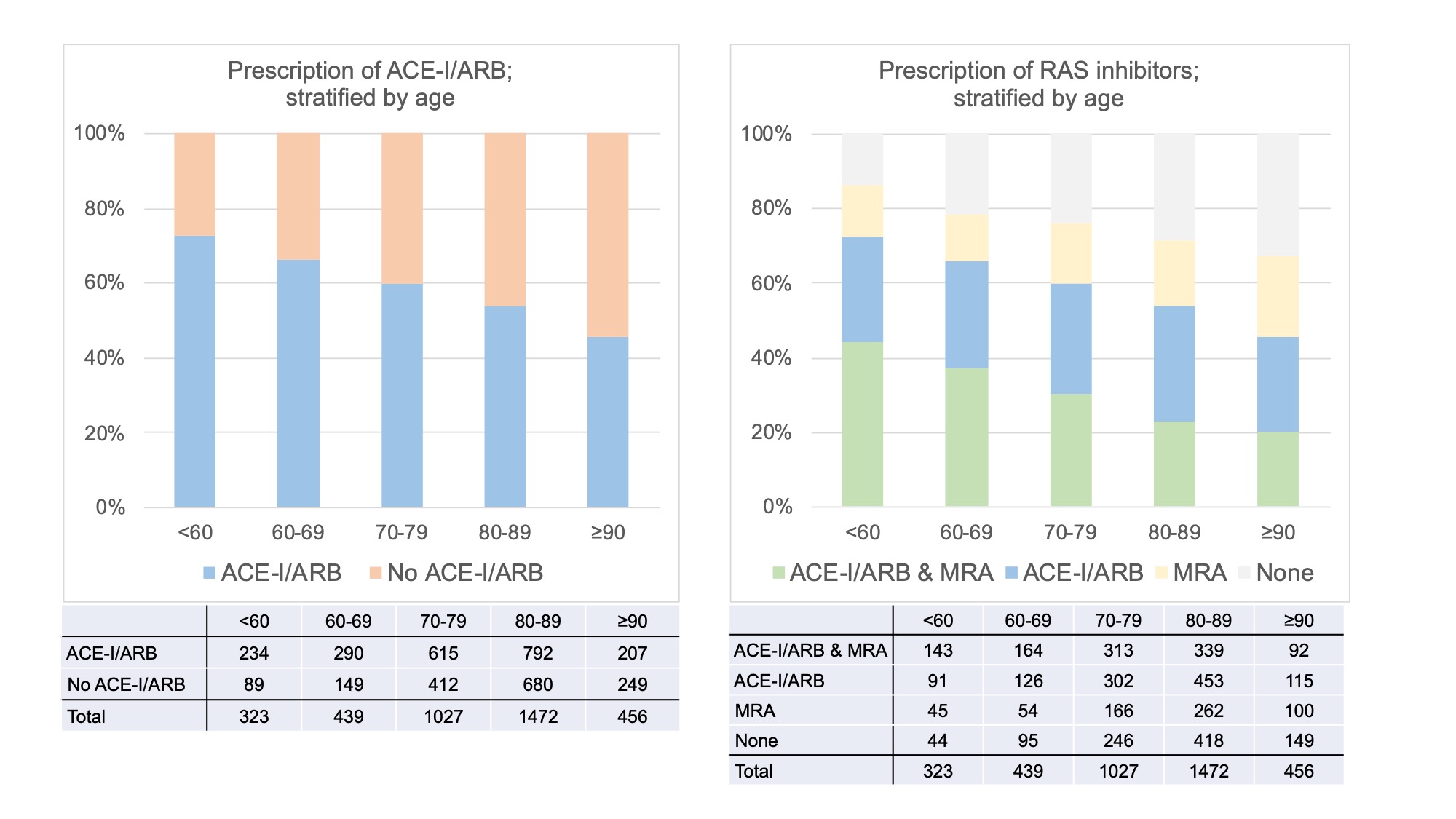


**B) Stratified by eGFR.**


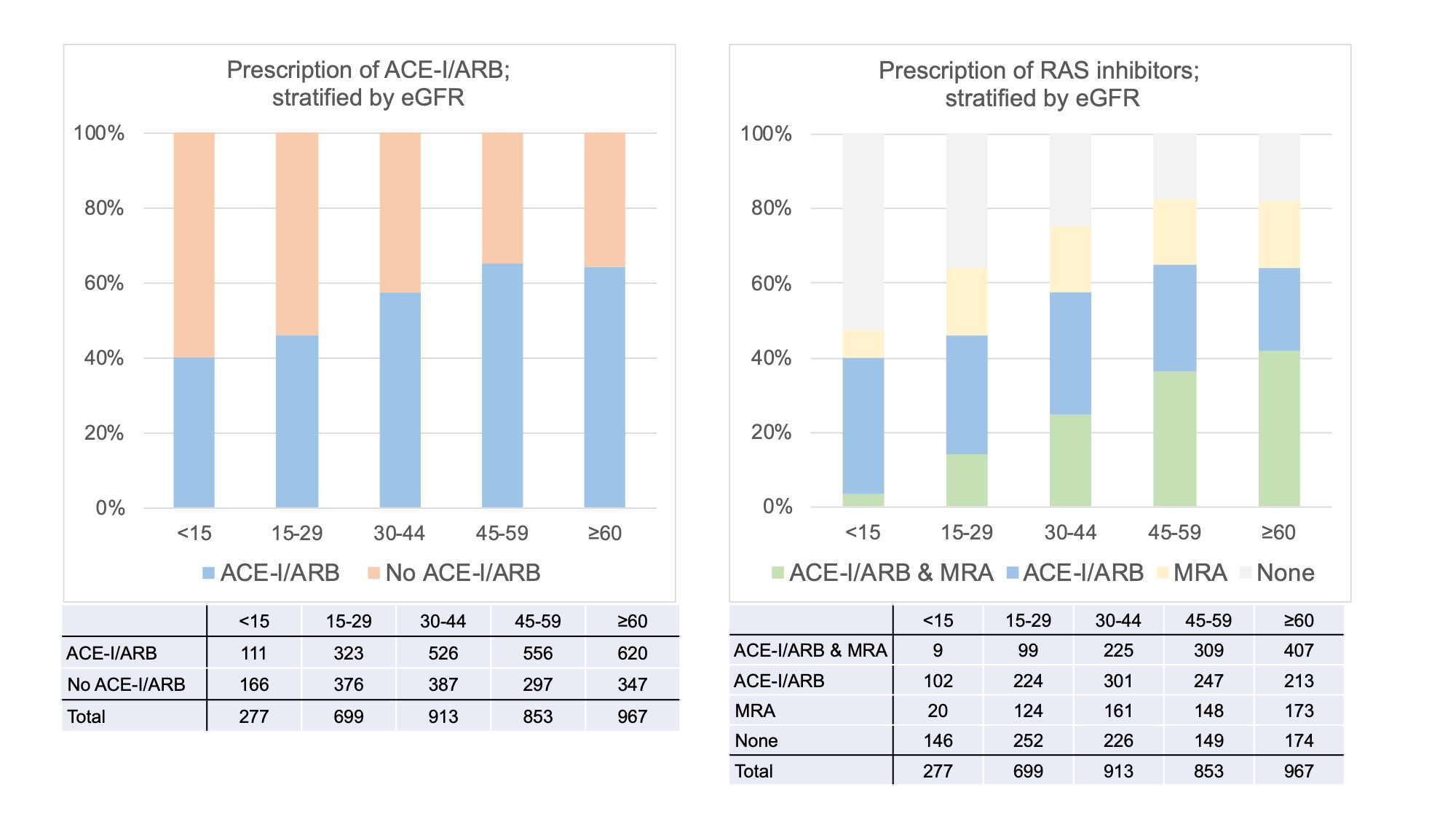


**C) Stratified by systolic BP at presentation.**


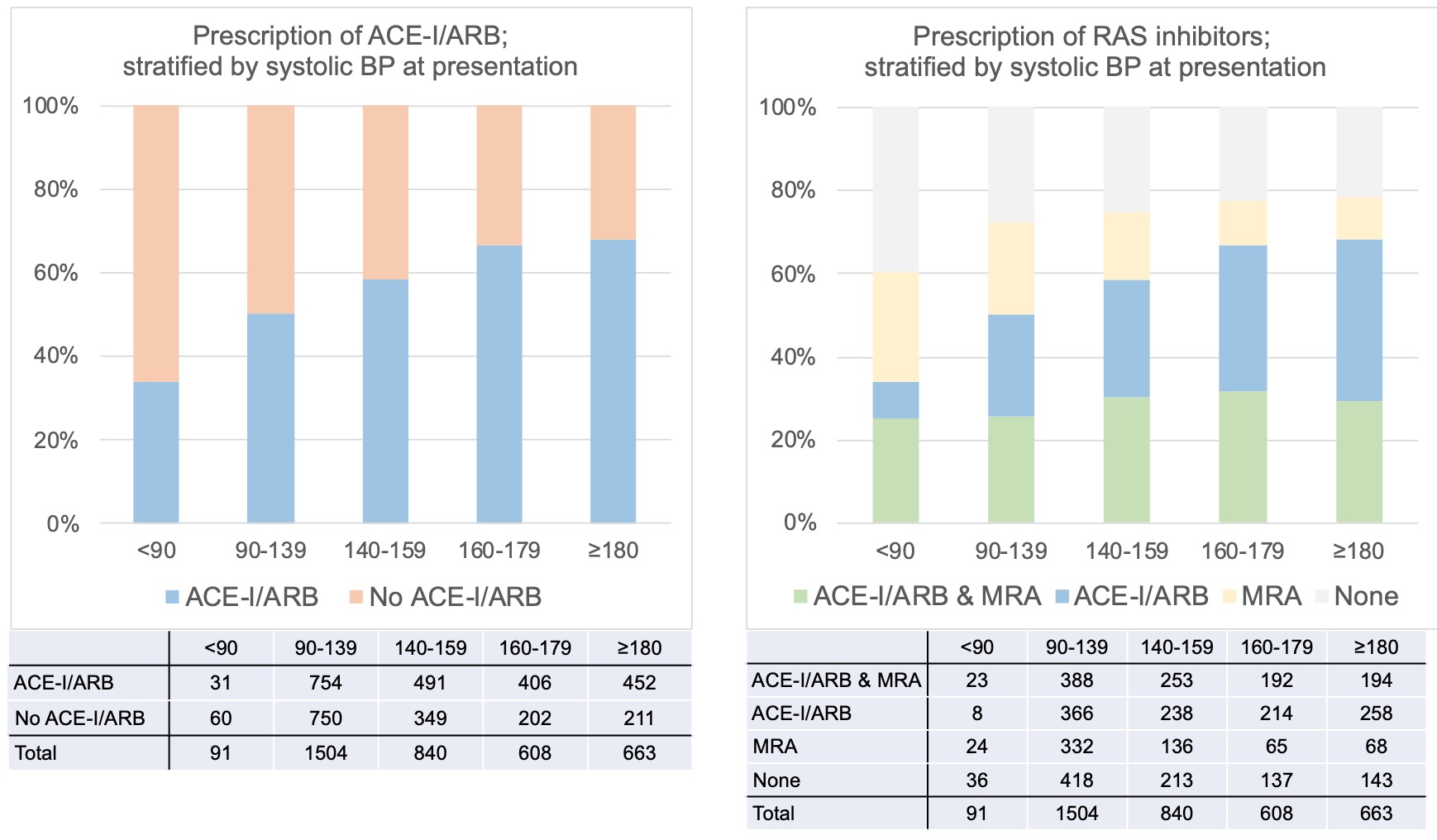


ACE-I, angiotensin-converting-enzyme inhibitors; ARB, angiotensin receptor blockers BP, blood pressure; eGFR, estimated glomerular filtration rate; LVEF, left ventricular ejection fraction; MRA, mineralocorticoid receptor antagonists.
