# Supplementary figures and images for: Impact of left ventricular ejection fraction on the effect of renin-angiotensin system blockers after an episode of acute heart failure: From the KCHF Registry
Source: PLoS One. 2020 Sep 14;15(9):e0239100. doi: 10.1371/journal.pone.0239100 (PMC7489562; doi:10.1371/journal.pone.0239100)

**S3 Fig. Kaplan-Meier curves for the clinical outcomes according to the LVEF categories.**

**
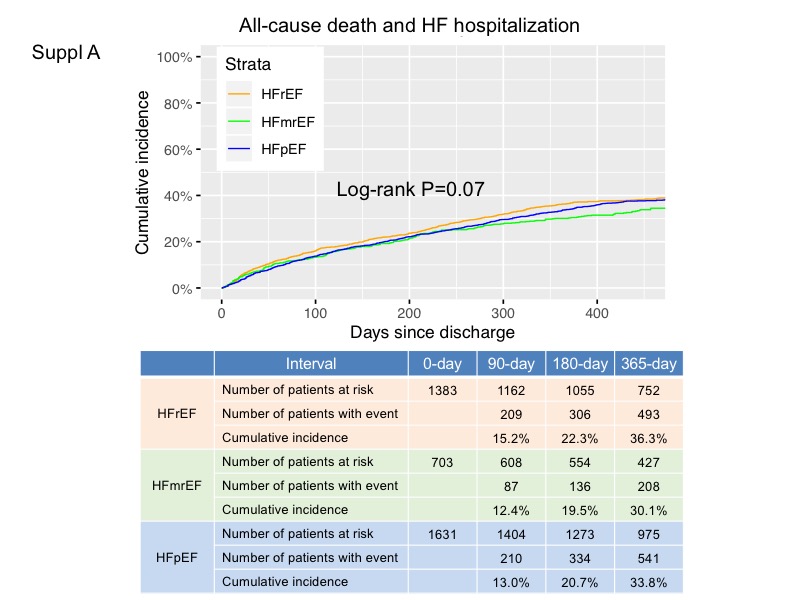
**

**
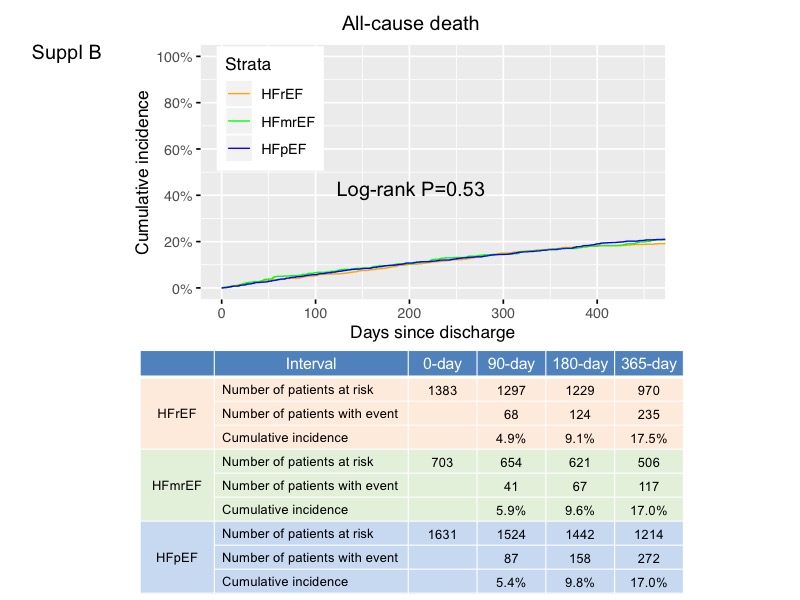
**

**
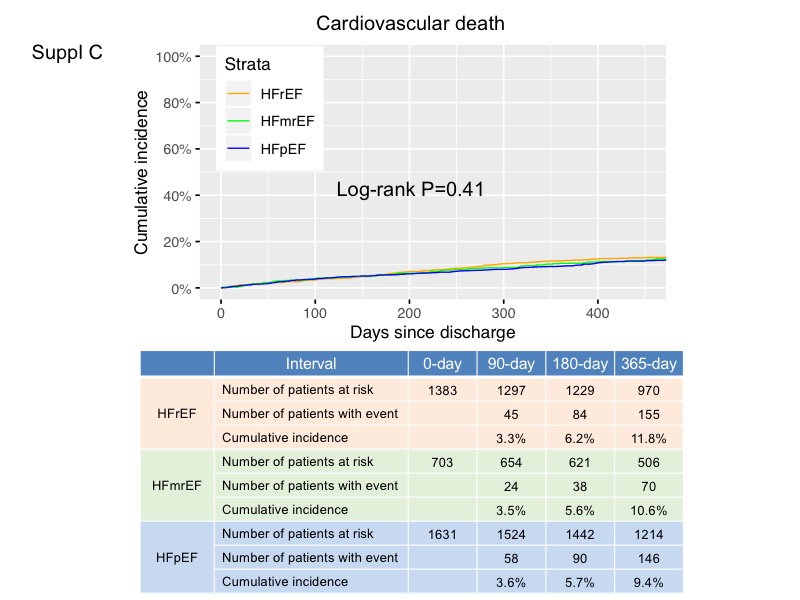
**

**
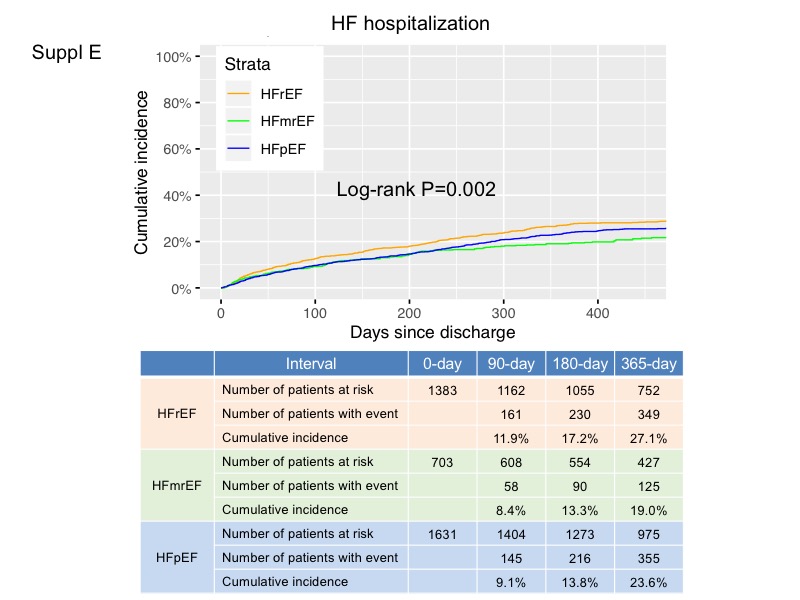
**

Supplement: S3 Fig — (DOCX) [file pone.0239100.s010.docx]
