# Supplementary material for: Impact of left ventricular ejection fraction on the effect of renin-angiotensin system blockers after an episode of acute heart failure: From the KCHF Registry
Source: PLoS One. 2020 Sep 14;15(9):e0239100. doi: 10.1371/journal.pone.0239100 (PMC7489562; doi:10.1371/journal.pone.0239100)
Supplement: S4 Fig — (DOCX) [file pone.0239100.s011.docx]

**S4 Fig: Propensity-score matching analysis and summarized results.**

In each ejection fraction (EF) stratum, we developed a propensity-score (PS) for the choice of angiotensin-converting enzyme inhibitors (ACE-I) or angiotensin receptor blockers (ARB) as a discharge medication. We used the logistic regression models with the use of ACE-I/ARB as a dependent variable and the 15 variables as independent variables, which were considered as clinically relevant to ACE-I/ARB prescription (listed in the Supplementary Appendix 5-7). Missing data of binary variables were considered as negative values. To create PS-matched cohorts, the patients with ACE-I/ARB were matched to those without ACE-I/ARB on the PSs by the nearest neighbor matching technique with a caliper of width equal to 0.2 of the standard deviation of the PS in each EF stratum. We constructed Cox proportional hazard models to estimate hazard ratios (HR) and their 95% confidence intervals (CI) of those with ACE-I/ARB relative to those without ACE-I/ARB for each clinical outcome measure.

The patient flowchart and the results are shown below. The risks of the use of ACE-I/ARB relative to the non-use for the primary outcome measure were in line with the main analysis; lower risks of the use of ACE-I/ARB relative to the non-use for the primary outcome measure were seen in HFrEF and HFmrEF, but not in HFpEF, although the small number of the matched population resulted in wide range of 95% confidence interval in the HFmrEF group. Other results in the PS matching analysis were also consistent with the main analysis for all the clinical outcomes except heart failure hospitalization in the HFrEF group.

**Patient flowchart**


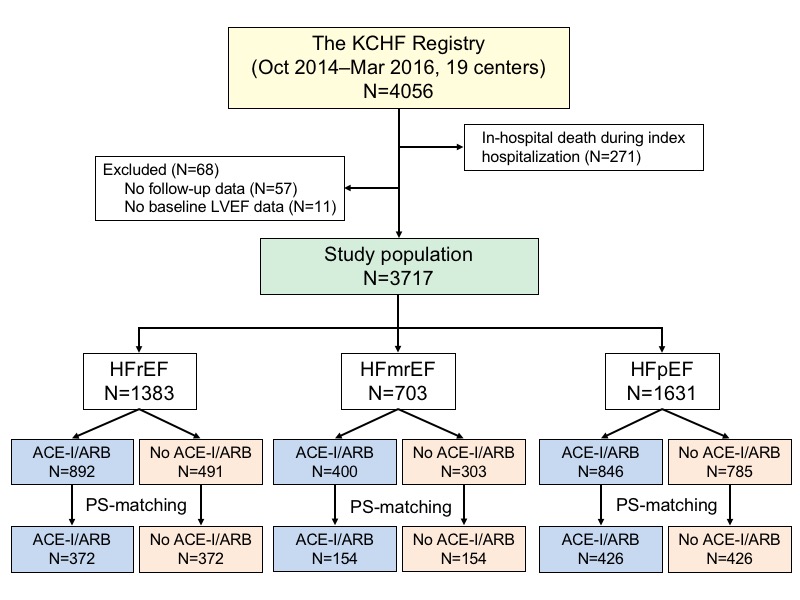


**The Kaplan-Meier curves for the primary endpoint in the PS-matched cohorts stratified by the LVEF categories; ACE-I/ARB group versus no ACE-I/ARB group.**

ACE-I, angiotensin-converting enzyme inhibitors; ARB, angiotensin receptor blockers; CI, confidence interval; HFmrEF, heart failure with mid-range ejection fraction; HFpEF, heart failure with preserved ejection fraction; HFrEF, heart failure with reduced ejection fraction.

**Effects of the use of ACE-I/ARB on clinical outcomes according to the LVEF categories in the propensity score-matched cohorts.**

|  | ACE-I/ARB* | No ACE-I/ARB* | HR (95% CI) | P value |
| --- | --- | --- | --- | --- |
| Primary outcome measure (all-cause death + HF hospitalization) | | | | |
| HFrEF | 128 (35.0%) | 148 (40.7%) | 0.79 (0.64–0.99) | 0.037 |
| HFmrEF | 38 (25.7%) | 50 (32.6%) | 0.73 (0.50–1.07) | 0.10 |
| HFpEF | 138 (33.0%) | 137 (33.2%) | 0.98 (0.79–1.21) | 0.86 |
| All-cause death | |  |  |  |
| HFrEF | 54 (15.0%) | 79 (21.9%) | 0.70 (0.52–0.95) | 0.02 |
| HFmrEF | 16 (10.9%) | 35 (23.0%) | 0.59 (0.35–0.96) | 0.036 |
| HFpEF | 70 (16.8%) | 79 (19.1%) | 0.79 (0.60–1.05) | 0.11 |
| HF hospitalization | |  |  |  |
| HFrEF | 99 (28.1%) | 92 (27.1%) | 0.97 (0.74–1.26) | 0.80 |
| HFmrEF | 23 (16.2%) | 29 (20.5%) | 0.72 (0.44–1.18) | 0.19 |
| HFpEF | 94 (23.7%) | 80 (21.1%) | 1.15 (0.88–1.52) | 0.30 |
| Cardiovascular death + HF hospitalization | |  |  |  |
| HFrEF | 115 (31.9%) | 120 (34.0%) | 0.87 (0.69–1.10) | 0.25 |
| HFmrEF | 34 (23.5%) | 40 (26.8%) | 0.76 (0.50–1.16) | 0.20 |
| HFpEF | 114 (27.9%) | 106 (26.7%) | 1.03 (0.81–1.30) | 0.82 |
| Cardiovascular death | |  |  |  |
| HFrEF | 35 (9.9%) | 49 (14.1%) | 0.73 (0.50–1.05) | 0.09 |
| HFmrEF | 12 (8.4%) | 23 (15.7%) | 0.58 (0.31–1.08) | 0.09 |
| HFpEF | 37 (9.2%) | 46 (11.5%) | 0.71 (0.48–1.04) | 0.08 |

*Presented as the number of patients with each clinical event (cumulative 1-year incidence estimated by the Kaplan-Meier method). Hazard ratios represent the risks of the use of ACE-I/ARB relative to the nonuse for the clinical outcomes.

ACE-I, angiotensin-converting enzyme inhibitors; ARB, angiotensin receptor blockers; HF, heart failure; HFmrEF, heart failure with mid-range ejection fraction; HFpEF, heart failure with preserved ejection fraction; HFrEF, heart failure with reduced ejection fraction; HR, hazard ratio.
